# Subunit P60 of phosphatidylinositol 3-kinase promotes cell proliferation or apoptosis depending on its phosphorylation status

**DOI:** 10.1371/journal.pgen.1009514

**Published:** 2021-04-26

**Authors:** Yu-Qin Di, Yu-Meng Zhao, Ke-Yan Jin, Xiao-Fan Zhao

**Affiliations:** Shandong Provincial Key Laboratory of Animal Cells and Developmental Biology, School of Life Sciences, Shandong University, Qingdao, China; University of Kentucky, UNITED STATES

## Abstract

The regulatory subunits (P60 in insects, P85 in mammals) determine the activation of the catalytic subunits P110 in phosphatidylinositol 3-kinases (PI3Ks) in the insulin pathway for cell proliferation and body growth. However, the regulatory subunits also promote apoptosis via an unclear regulatory mechanism. Using *Helicoverpa armigera*, an agricultural pest, we showed that *H*. *armigera* P60 (HaP60) was phosphorylated under insulin-like peptides (ILPs) regulation at larval growth stages and played roles in the insulin/ insulin-like growth factor (IGF) signaling (IIS) to determine HaP110 phosphorylation and cell membrane translocation; whereas, HaP60 was dephosphorylated and its expression increased under steroid hormone 20-hydroxyecdysone (20E) regulation during metamorphosis. Protein tyrosine phosphatase non-receptor type 6 (HaPTPN6, also named tyrosine-protein phosphatase corkscrew-like isoform X1 in the genome) was upregulated by 20E to dephosphorylate HaP60 and HaP110. 20E blocked HaP60 and HaP110 translocation to the cell membrane and reduced their interaction. The phosphorylated HaP60 mediated a cascade of protein phosphorylation and forkhead box protein O (HaFOXO) cytosol localization in the IIS to promote cell proliferation. However, 20E, via G protein-coupled-receptor-, ecdysone receptor-, and HaFOXO signaling axis, upregulated HaP60 expression, and the non-phosphorylated HaP60 interacted with phosphatase and tensin homolog (HaPTEN) to induce apoptosis. RNA interference-mediated knockdown of *HaP60* and *HaP110* in larvae repressed larval growth and apoptosis. Thus, HaP60 plays dual functions to promote cell proliferation and apoptosis by changing its phosphorylation status under ILPs and 20E regulation, respectively.

## Author summary

The regulatory subunits of phosphatidylinositol 3-kinases (PI3Ks) play very important roles in various pathways by promoting cell proliferation or apoptosis. However, the upstream regulatory mechanism of their opposite functions is unclear. Using a seriously agricultural pest *Helicoverpa armigera* as a model, we show that ILPs induce HaP60 phosphorylation to increase HaP110 phosphorylation and cell membrane location to promote cell proliferation. 20E promotes HaP60 and HaP110 dephosphorylation that resulted in the cytosol localization and inhibition of PI3K activity. Moreover, 20E elevates HaP60 expression to promote apoptosis. Our study revealed that HaP60 plays dual functions to regulate cell proliferation and apoptosis by changing its phosphorylated status.

## Introduction

Phosphatidylinositol 3-kinases (PI3Ks) play very important roles in various pathways of cellular responses to the extracellular signals of insulin and insulin-like growth factor (IGF) [1]. PI3Ks are composed of different P110 catalytic subunits and different P85 (P60 in insects) regulatory subunits in mammals [2]. In insulin signaling, the insulin receptor (INSR) is autophosphorylated by insulin binding-induced conformational change, which in turn phosphorylates insulin receptor substrate (IRS) and P85, thus resulting in the recruitment of the P85-P110 complex to the plasma membrane [3], during which P110 is autophosphorylated [4]. P110 phosphorylates phosphatidylinositol 4,5-bisphosphate (PIP2) to form phosphatidylinositol 3,4,5-trisphosphate (PIP3) in the cell membrane, which attracts protein kinase B/AKT to the cell membrane, where it is phosphorylated by the cell membrane located phosphoinositide-dependent kinase (PDK) [5]. The phosphorylated AKT promotes cell proliferation in physiological and oncological conditions and phosphorylates forkhead box protein O (FOXO) to prevent it from entering the nucleus to play a transcription factor role in apoptosis [6]. Phosphatase and tensin homolog (PTEN) reverses the function of PI3K by dephosphorylating PIP3 [7]. The regulatory subunits (P85α, P85β, and P55γ in mammals) can bind and stabilize P110s [8]. However, regulatory subunits have different functions in different cells. The regulatory subunit P85β is a tumor driver in melanoma and breast cancer, whereas P85α functions oppositely as a tumor suppressor in breast cancer [9]. P55γ is necessary for ovarian cell proliferation [10]; however, P55γ suppresses vascular smooth muscle cell proliferation [11]. The mechanism of the contradictory functions of regulatory subunits is unclear.

Insect growth and metamorphosis are mainly regulated by insulin-like peptides (ILPs) and the steroid hormone 20-hydroxyecdysone (20E) in different insects [12, 13]. ILPs regulate the growth rate by promoting cell growth and proliferation, and 20E determines growth termination and promotes apoptosis for metamorphosis [14]. ILPs, via insulin-like receptor (InR), promote cell proliferation and body growth [15, 16]. Meanwhile, it promotes prothoracic gland (PG) growth to produce more ecdysone [17], which is converted to 20E as a systemic signal to end the larval growth and initiate metamorphosis [18]. 20E enters its target cells through ecdysone importer (EcI), a membrane transporter, and binds to ecdysone receptor (EcR)-ultraspiracle (USP) transcription complex to activate transcription of multiple genes that are involved in molting and metamorphosis [19–22]. Although the previous studies showed that 20E counteracts insulin/IGF signaling (IIS) activity during development [23], the mechanism by which 20E regulates PI3K to antagonize IIS is poorly understood.

In *H*. *armigera*, a lepidopteran insect and a serious agricultural pest, class IA PI3K consists of a single regulatory subunit gamma, named HaP60, showing an equal degree of similarity to mammalian P85α, P85β, and P55γ; and a single catalytic subunit delta, named HaP110, being homologous to mammalian class IA PI3Ks. This is consistent with *Drosophila melanogaster* [24], which presents a simple PI3K model to address the roles and regulatory mechanism of P60 by ILPs and steroid hormone. Therefore, using *H*. *armigera*, we demonstrated that HaP60 functions as a phosphorylated form to promote cell proliferation by interacting with HaP110 under ILPs regulation, and functions as a non-phosphorylated form to promote apoptosis by interacting with HaPTEN under 20E regulation.

## Results

### HaP60 and HaP110 show variations in phosphorylation and expression levels during insect development

**Fig 1:**
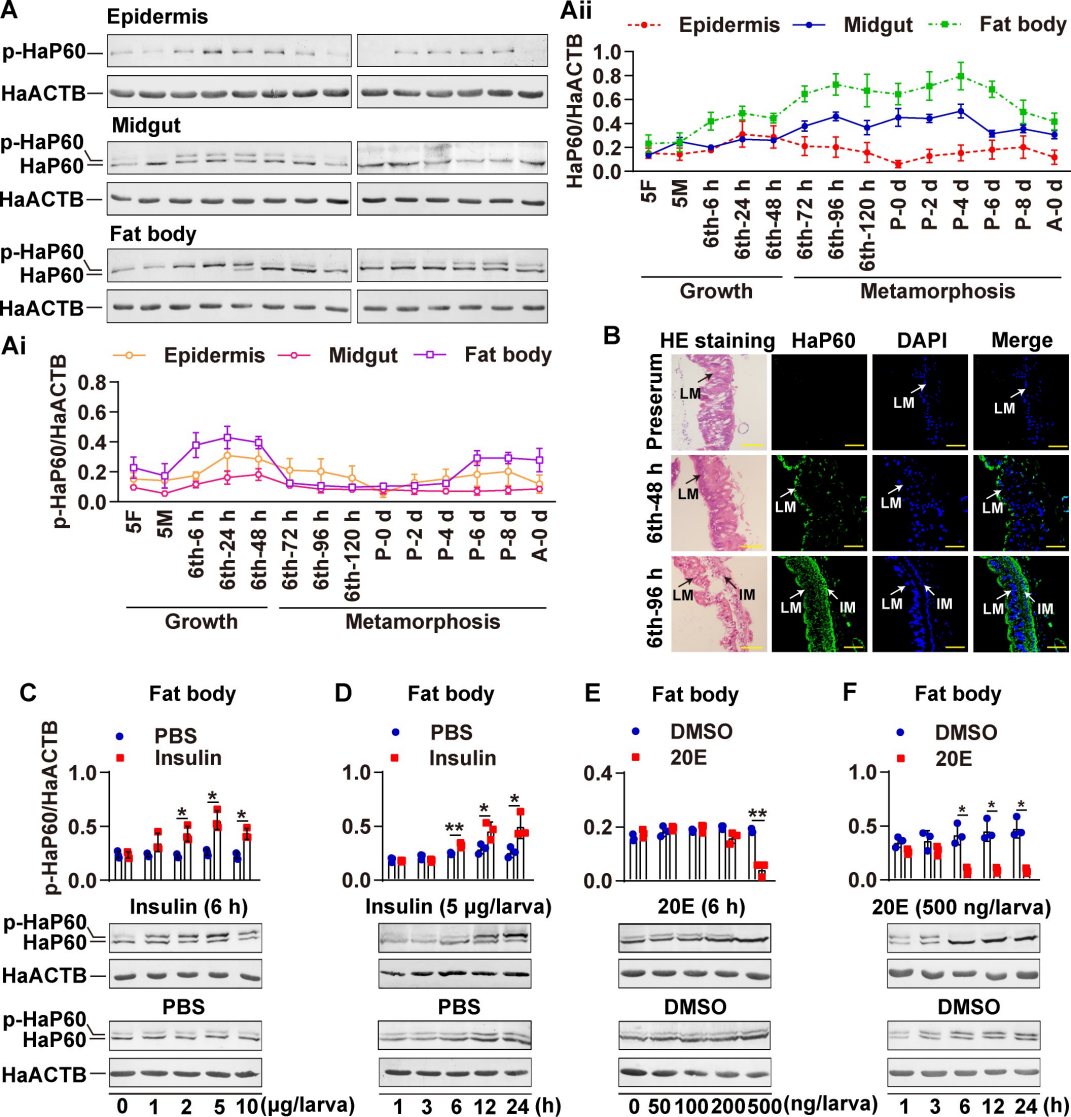
20E promoted HaP60 dephosphorylation and expression. **A.** Western blotting detection of the expression profiles of HaP60 from the fifth instar to adult in different tissues using anti-HaP60 antibodies. 5F, fifth instar feeding; 5M, fifth instar molting; 6th-6 h-120 h, sixth instar 6 h-120 h; P 0 d-P 8 d, pupa 0 d-8 d; A 0 d, adult 0 d. **Ai.** The quantitation of phosphorylated HaP60 in A. **Aii.** The quantitation of HaP60 in A. **B.** HE staining and immunohistochemistry showing HaP60’s location in the midgut of 6th-48 h and 6th-96 h. Green, HaP60 detected using anti-HaP60 antibodies; blue, nuclei stained using DAPI; merge, overlapped with green and blue; IM, imaginal midgut; LM, larval midgut. Bar, 20 μm. **C and D.** Insulin elevated HaP60 phosphorylation in a dose and time dependent manner, respectively. **E and F.** 20E promoted HaP60 dephosphorylation and expression in a dose and time dependent manner, respectively. All experiments were repeated at least three times. Every column contained three blue dots or red squares represents the quantitation of independently repeated three experiments. *P < 0.05 and **P < 0.01 using two-tailed Student’s *t*-test. The bars indicate mean ± SD.

To investigate the function of HaP60 in *H*. *armigera* development, we detect its developmental expression profiles using rabbit polyclonal antibodies against *H*. *armigera* HaP60 (anti-HaP60 antibodies) prepared in our laboratory, and the specificities of the antibodies were examined (S1A Fig). Western blotting showed that HaP60 appeared as a single band in the epidermis, but HaP60 appeared as two bands in the midgut and fat body (S1B Fig), and the band in the epidermis and the upper band in the midgut and fat body were identified as a phosphorylated form of HaP60 using lambda protein phosphatase (λPPase) treatment (S1C Fig). The phosphorylated HaP60 was increased at the sixth instar feeding stages and decreased at the metamorphic stages, accompanying increased expression levels at the metamorphosis compared with the fifth instar feeding stages and sixth instar feeding stages in the midgut and fat body (Fig 1A–Aii). Hematoxylin and eosin (HE) staining and immunohistochemistry showed that HaP60 was localized in both the larval midgut and imaginal midgut (Fig 1B). These results suggested that HaP60 plays roles in larval and adult tissues as a phosphorylated form in the feeding stages and as a non-phosphorylated form during metamorphosis.

To address the hormonal regulation of HaP60 phosphorylation and expression, insulin or 20E were injected into the hemocoel of the sixth instar 6 h (6th-6 h) larvae, separately. Insulin promoted HaP60 phosphorylation, with little effect on its expression, in a dose- and time-dependent manner, compared with that in the phosphate-buffered saline (PBS) control. In contrast, 20E induced HaP60 dephosphorylation and expression in a dose- and time-dependent manner, compared with that in the dimethyl sulfoxide (DMSO) control (Fig 1C–1F). These results revealed that ILPs induce HaP60 phosphorylation and 20E induces HaP60 dephosphorylation but increases its expression.

**Fig 2:**
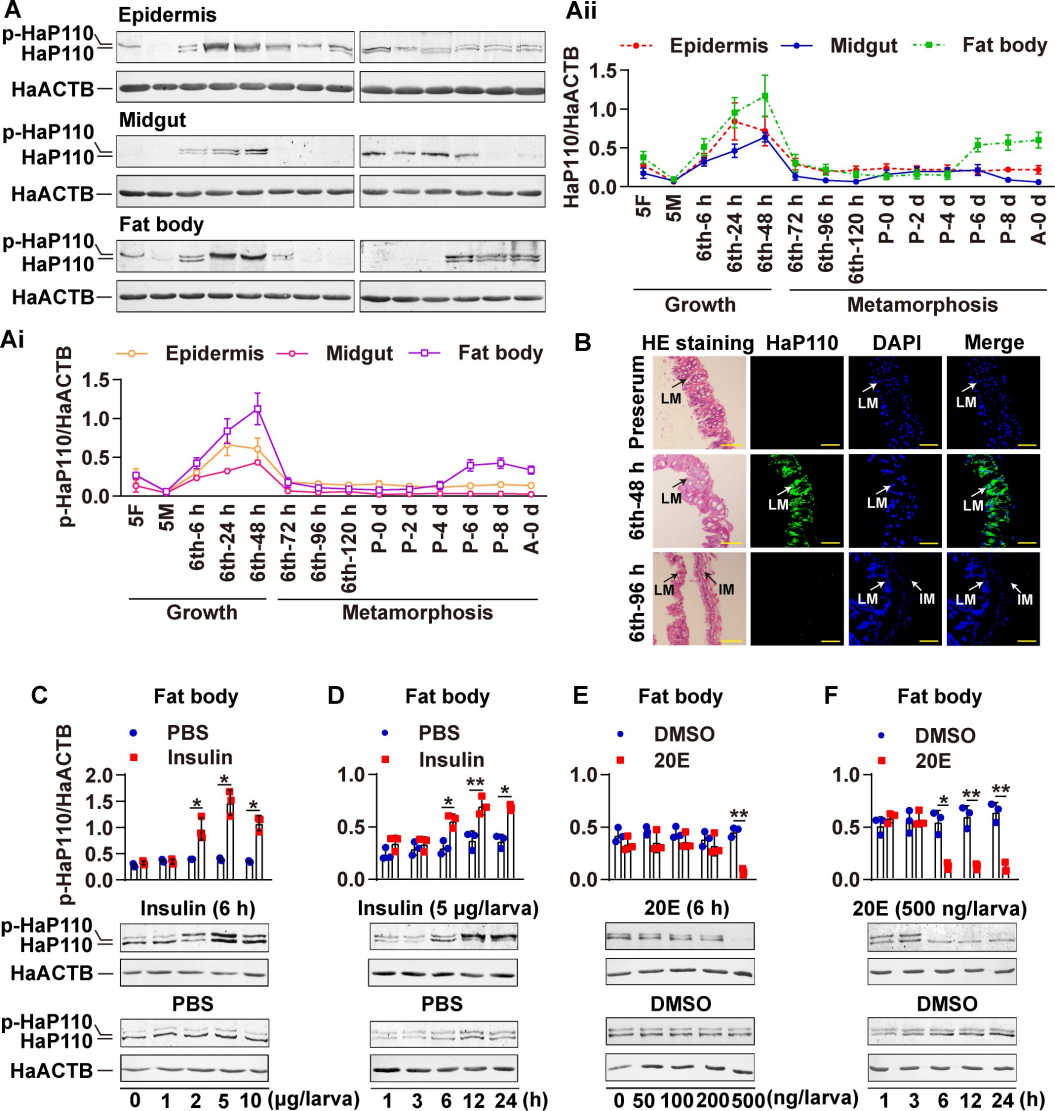
20E repressed HaP110 phosphorylation and expression. **A.** Western blotting detection of the expression profiles of HaP110 from the fifth instar to adult in different tissues using anti-HaP110 antibodies. 5F, fifth instar feeding; 5M, fifth instar molting; 6th-6 h-120 h, sixth instar 6 h-120 h; P 0 d-P 8 d, pupa 0 d-8 d; A 0 d, adult 0 d. **Ai.** The quantitation of phosphorylated HaP110 in A. **Aii.** The quantitation of HaP110 in A. **B.** HE staining and immunohistochemistry showed HaP110’s location in the midgut of 6th-48 h and 6th-96 h. Green, HaP110 detected using anti-HaP110 antibodies; blue, nuclei stained using DAPI; merge, overlapped with green and blue; IM, imaginal midgut; LM, larval midgut. Bar, 20 μm. **C and D.** Insulin elevated HaP110 phosphorylation and expression in a dose and time dependent manner, respectively. **E and F.** 20E increased HaP110 dephosphorylation but decreased HaP110 expression in a dose and time dependent manner. All experiments were repeated at least three times. Every column contained three blue dots or red squares represents the quantitation of independently repeated three experiments. *P < 0.05 and **P < 0.01 using two-tailed Student’s *t*-test. The bars indicate mean ± SD.

The expression profiles of HaP110 were also examined to determine its role in insect development using rabbit polyclonal antibodies against *H*. *arimgera* HaP110 (anti-HaP110 antibodies) prepared in our laboratory (S1 Fig). HaP110 appeared to be phosphorylated and showed high expression levels at the growth stage, with increased expression during the early pupal stage in the midgut. Furthermore, HaP110 was phosphorylation and had high expression levels at the growth stages and later pupal stage in the fat body (Fig 2A-Aii). HE staining and immunohistochemistry showed that HaP110 was mainly localized in the larval midgut at the 6th-48 h stage (Fig 2B). Insulin increased HaP110 expression and phosphorylation in a dose- and time-dependent manner, whereas 20E decreased HaP110 expression and phosphorylation in a dose- and time-dependent manner (Fig 2C–2F). Thus, HaP110 plays roles in different tissues during larval growth, and its expression and phosphorylation were regulated by ILPs and 20E, counteractively.

### 20E promotes dephosphorylation of HaP60 and HaP110 by upregulating HaPTPN6 expression

**Fig 3:**
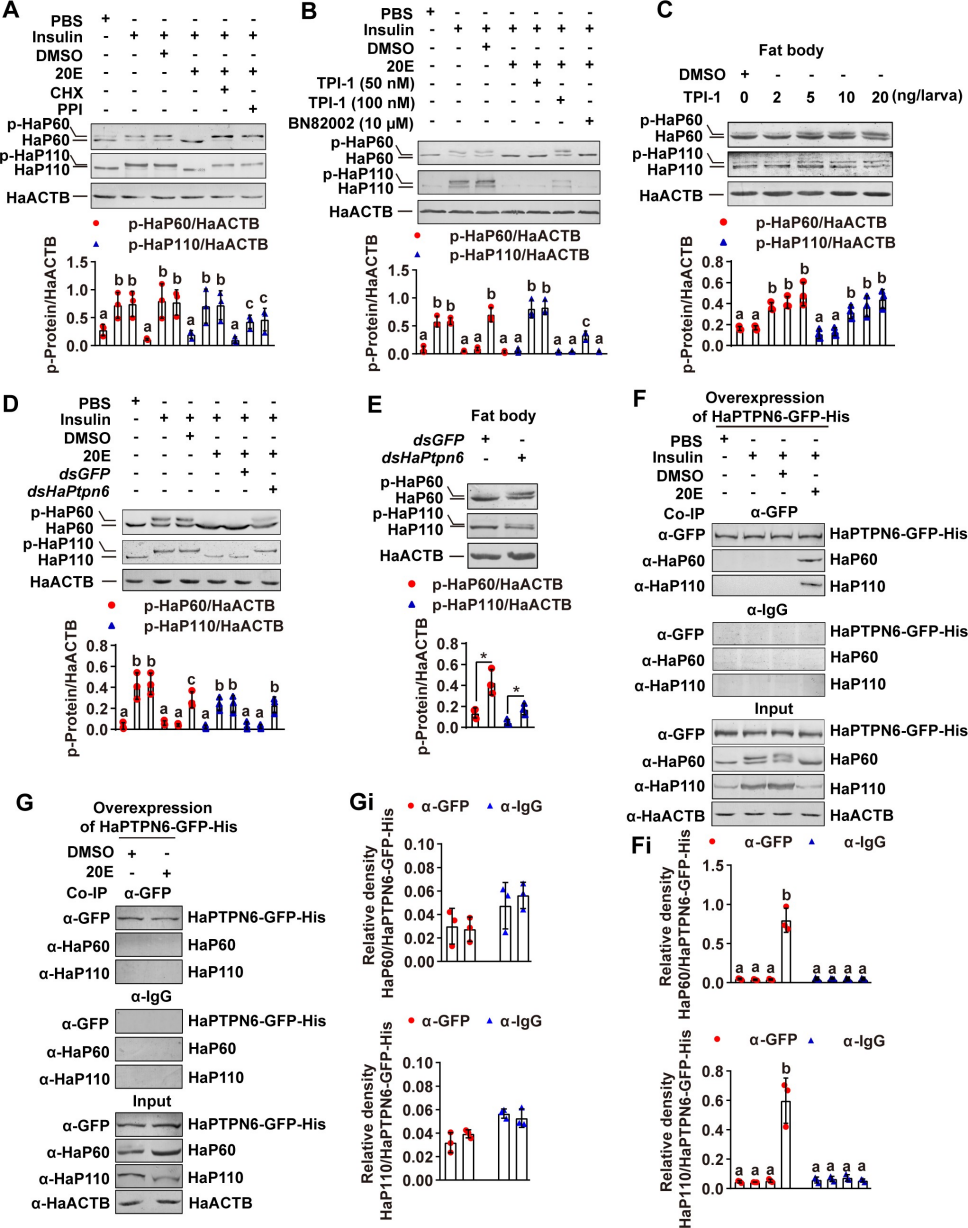
HaPTPN6 dephosphorylated HaP60 and HaP110. **A.** CHX and PPI inhibited 20E induced-HaP60 and HaP110 dephosphorylation in HaEpi cells. CHX (50 μg/mL for 1 h); PPI (1% for 1 h); insulin (5 μg/mL for 1 h); 20E (5 μM for 6 h). **B.** TPI-1 inhibited 20E induced-HaP60 and HaP110 dephosphorylation in HaEpi cells. **C.** TPI-1 inhibited 20E induced-HaP60 and HaP110 dephosphorylation in the fat body of 6th-72 h larvae. **D.** *HaPtpn6* knockdown repressed 20E-induced HaP60 and HaP110 dephosphorylation in HaEpi cells. **E.** *HaPtpn6* knockdown repressed 20E-induced HaP60 and HaP110 dephosphorylation in the fat body of 6th-72 h larvae. **F.** 20E mediated the interaction of HaP60 and HaP110 with HaPTPN6 under insulin treatment. The anti-GFP antibody (α-GFP) immunoprecipitated overexpressed HaPTPN6-GFP-His in the cell lysate, co-immunoprecipitated HaP60 and HaP110 were detected using anti-HaP60 antibodies (α-HaP60) and anti-HaP110 antibodies (α-HaP110), respectively. Mouse IgG (α-IgG) was used as a negative control for antibody. **Fi.** Statistical analysis for F. **G.** 20E rarely promoted the interaction of HaP60 and HaP110 with HaPTPN6. The anti-GFP antibody (α-GFP) immunoprecipitated overexpressed HaPTPN6-GFP-His in the cell lysate, co-immunoprecipitated HaP60 and HaP110 were detected using anti-HaP60 antibodies (α-HaP60) and anti-HaP110 antibodies (α-HaP110), respectively. Mouse IgG (α-IgG) was used as a negative control for antibody. **Gi.** Statistical analysis for G. *P < 0.05 using two-tailed Student’s *t*-test or different lowercase letters indicate significant differences (*P* < 0.05) using one-way ANOVA. The bars indicate the mean ± SD.

To explore the mechanism of 20E-promoted dephosphorylation of HaP60 and HaP110, the protein translation inhibitor Cycloheximide (CHX) and phosphatase inhibitor (PPI) were added to HaEpi cells (a *Helicoverpa armigera* epidermal cell line), followed by insulin and 20E induction, respectively. The results showed that insulin induced HaP60 and HaP110 phosphorylation; however, 20E repressed HaP60 and HaP110 phosphorylation, and CHX and PPI maintained HaP60 and HaP110 phosphorylation, suggesting that 20E relies on a phosphatase to dephosphorylate HaP60 and HaP110 (Fig 3A). From the literature survey, the tyrosine phosphatase, protein tyrosine phosphatase non-receptor type 6 (PTPN6), was known to act with P85 to inhibit PI3K activity in mammalian cells [25]. We identified HaPTPN6 by BLAST (basic local alignment search tool) of the genome of *H*. *armigera* using mammalian PTPN6 (S2 Fig). Therefore, the specific inhibitor of PTPN6, tyrosine phosphatase inhibitor 1 (TPI-1), was used to examine the role of HaPTPN6 in 20E-mediated dephosphorylation of HaP60 and HaP110. BN82002, an inhibitor of the cell division cycle 25 (CDC25), as control of TPI-1. TPI-1 treatment repressed 20E-mediated dephosphorylation of HaP60 and HaP110 in HaEpi cells and the larval fat body (Fig 3B and 3C), but BN82002 did not. After knockdown of *HaPtpn6* in HaEpi cells, 20E could not induce dephosphorylation of HaP60 and HaP110 (Fig 3D). Quantitative real-time reverse transcription PCR (qRT-PCR) showed *HaPtpn6* was knocked down in the HaEpi cells without off-target effects on another protein phosphatase gene, *HaPtpn11* (S3 Fig). Knockdown of *HaPtpn6* in larvae by feeding third instar larvae with *Escherichia coli* HT115 (*E*. *coli* HT115) that expressed *dsHaPtpn6* also maintained HaP60 and HaP110 phosphorylation in the larval fat body at the 6th-72 h stage, compared with *dsGFP* (Fig 3E). Meanwhile, Phenotype analysis showed *HaPtpn6* knockdown resulted in 53% early pupation than 9% early pupation in *dsGFP*, and advanced pupation time for 13 h (S4A–S4C Fig), but the average weight of pupa had no difference between *dsHaPtpn6* and *dsGFP* (S4D Fig). QRT-PCR showed *HaPtpn6* was knocked down in the larvae without off-target effects on *HaPtpn11* (S4E Fig). The midgut appeared red, an indication of programmed cell death (PCD), at 8 d post-feeding *dsHaPtpn6*, whereas, the larval midgut was not red and did not separate from the imaginal midgut at the same time after feeding with *dsGFP* (S4F and S4G Fig). Moreover, the larval fat body retained complete after feeding with *dsGFP*-fed control at 8 d, whereas the larval fat body started to decompose in the *dsHaPtpn6*-fed larvae (S4H Fig). These results suggest HaPTPN6 is necessary for HaP60 and HaP110 dephosphorylation and initiation of metamorphosis. Co-immunoprecipitation (Co-IP) using anti-GFP antibody that recognized the overexpressed HaPTPN6-GFP-His in HaEpi cells demonstrated that 20E promoted the interaction of HaP60 and HaP110 with HaPTPN6 after insulin induction (Fig 3F and 3Fi), but 20E treatment alone hardly promoted the interaction of HaP60 and HaP110 with HaPTPN6 (Fig 3G and 3Gi), suggesting HaPTPN6 binds to HaP60/HaP110 relying on their phosphorylation status. These data suggested that HaPTPN6 directly dephosphorylates HaP60 and HaP110 under 20E regulation.

In addition, *HaPtpn6* was highly expressed in the fat body during metamorphosis (S5A Fig). 20E upregulated *HaPtpn6* expression in a dose and time-dependent manner via ecdysone responsive G protein-coupled-receptors (ErGPCRs), EcR, and USP1 (S5B–S5I Fig). 20E promoted EcR binding to the ecdysone receptor element (EcRE), 5′-_-1723_GAAGTCAATGAAATT_-1709_-3′, in the promoter of *HaPtpn6* (S5J Fig), revealing the signaling axis by which 20E upregulates *HaPtpn6* transcription.

### 20E blocks ILP-induced cell membrane translocation and decreases the interaction of HaP60 and HaP110 by dephosphorylation of HaP60 and HaP110

**Fig 4:**
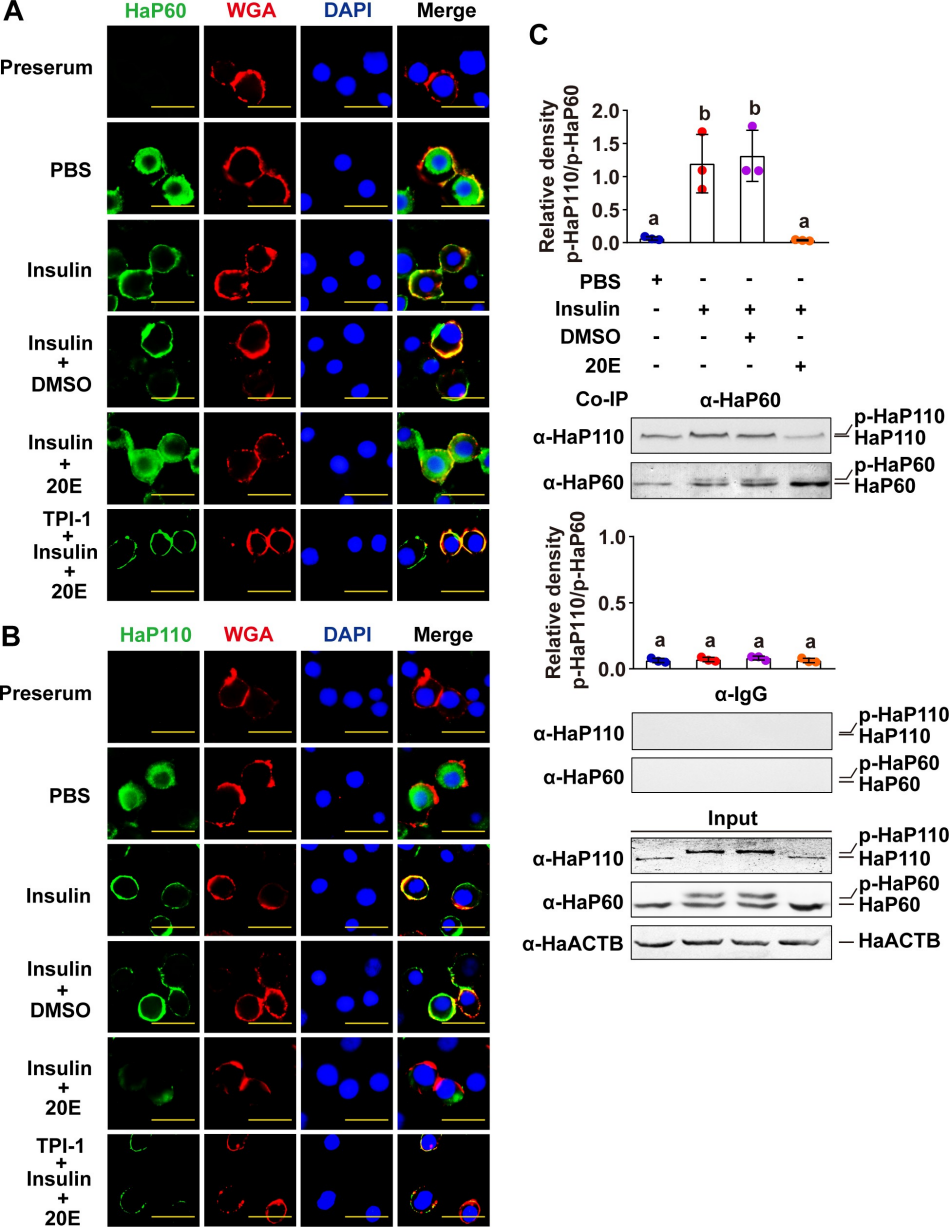
20E repressed ILP-mediated cell membrane translocation of HaP60 and HaP110, dephosphorylated HaP60 and HaP110, and decreased their interaction. **A and B.** Immunocytochemistry showed that 20E repressed insulin-mediated HaP60 and HaP110 cell membrane localization in HaEpi cells by dephosphorylating HaP60 and HaP110. Insulin (5 μg/mL for 1 h); PBS as control of insulin; 20E (5 μM for 6 h); DMSO as control of 20E; TPI-1 (100 nM). Green, HaP60 or HaP110; red, cell membrane stained with WGA; blue, nuclei stained with DAPI; merge, overlapped green, blue, and red fluorescence. Bar, 50 μm. **C.** Co-IP assay showed that 20E decreased the HaP60–HaP110 interaction and induced dephosphorylation in cells. Anti-HaP60 antibodies (α-HaP60) immunoprecipitated HaP60 and HaP110, followed by detection using anti-HaP60 antibodies (α-HaP60) and anti-HaP110 antibodies (α-HaP110), respectively. Rabbit IgG (α-IgG) was used as a negative control for anti-HaP60 antibodies. Different lowercase letters indicate significant differences (*P* < 0.05) using one-way ANOVA. The bars indicate the mean ± SD.

To address the consequence of HaP60 and HaP110 dephosphorylation, we observed the subcellular localization of HaP60 and HaP110. Antibodies detection showed that HaP60 and HaP110 were localized in the cytosol in the PBS control in HaEpi cells. Insulin induced HaP60 and HaP110 to transfer to the cell membrane from the cytosol; however, 20E blocked the insulin-induced cell membrane translocation of HaP60 and HaP110, compared with the DMSO control, nevertheless, 20E hardly blocked the subcellular localization of HaP60 and HaP110 upon TPI-1 treatment (Fig 4A and 4B). The Co-IP assay showed that insulin increased the phosphorylation and interaction of the HaP60 and HaP110, but whereas 20E decreased the interaction between HaP60 and HaP110 when they were dephosphorylated (Fig 4C), suggesting that 20E inhibits the interaction between HaP60 and HaP110, and cell membrane translocation, by dephosphorylating HaP60 and HaP110 via HaPTPN6. 20E also repressed HaP110 expression; therefore, the signal for HaP110 was weak under 20E treatment.

### HaP60 promotes cell proliferation under ILP regulation

**Fig 5:**
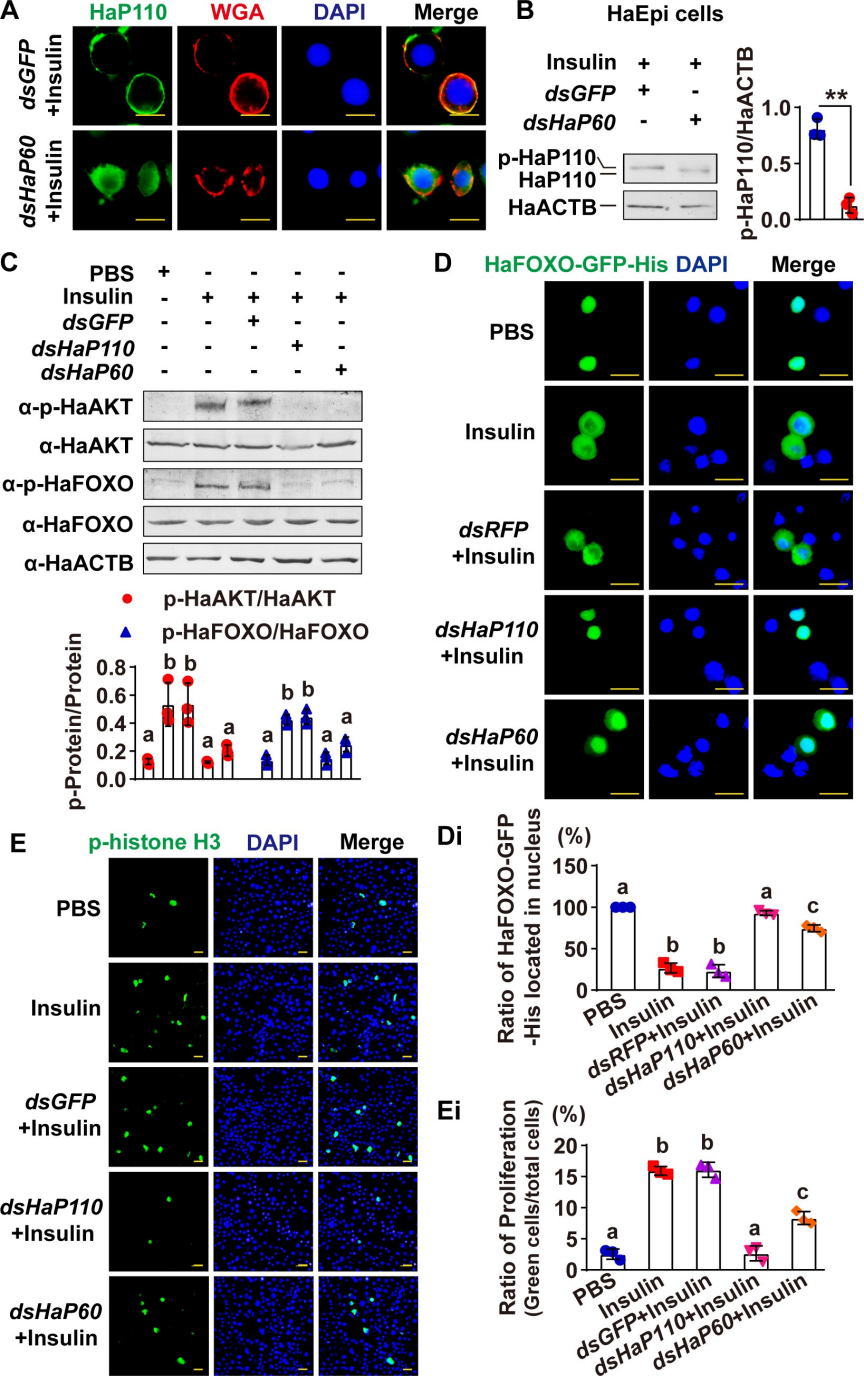
*HaP60* knockdown repressed ILP-mediated actions. **A.** *HaP60* knockdown repressed insulin-mediated HaP110 cell membrane translocation. Green, HaP110; red, cell membrane stained with WGA; blue, nuclei stained with DAPI; merge, overlapped green, blue, and red fluorescence. Bar, 20 μm. **B.** *HaP60* knockdown repressed insulin-mediated HaP110 phosphorylation. **C.** *HaP60* and *HaP110* knockdown repressed insulin-mediated HaAKT and HaFOXO phosphorylation. **D.** *HaP60* and *HaP110* knockdown repressed insulin-mediated HaFOXO cytosolic localization. HaFOXO-GFP-His was overexpressed in HaEpi cells. Insulin (5 μg/mL, for 1 h); Green, HaFOXO-GFP-His; blue, nuclei stained with DAPI; merge, the overlapped green and blue fluorescence. Bar, 50 μm. **Di.** Statistical analysis of HaFOXO-GFP-His localization in the nuclei of D. **E.** *HaP60* or *HaP110* knockdown repressed insulin-induced cell proliferation. Green, phospho-histone H3 detected by anti-phospho-histone H3 antibodies; blue, nuclei stained with DAPI; merge, the overlapped green and blue fluorescence. Bar, 50 μm. **Ei.** Statistical analysis of phospho-histone H3 in E. **P < 0.01 using a two-tailed Student’s *t*-test or different lowercase letters indicate significant differences (*P* < 0.05) using one-way ANOVA. The bars indicate the mean ± SD.

To investigate the function of HaP60 in IIS, the HaP110 subcellular localization and subsequent protein phosphorylation cascade were explored via *HaP60* knockdown followed by insulin treatment. The results showed that *HaP60* knockdown partially blocked the insulin-induced cell membrane translocation of HaP110 (Fig 5A). Western blotting showed that HaP110 was not phosphorylated by insulin induction after *HaP60* knockdown (Fig 5B). *HaP60* knockdown decreased insulin-induced HaAKT and HaFOXO phosphorylation, which was similar to the results of *HaP110* knockdown (Fig 5C). *HaP60* and *HaP110* knockdown blocked the insulin-induced HaFOXO cytosolic localization, resulting in HaFOXO locating to the nucleus, compared with *dsRFP* (Fig 5D and 5Di). *HaP60* and *HaP110* knockdown decreased insulin-induced cell proliferation (Fig 5E and 5Ei). Western blotting and qRT-PCR showed that *HaP60* and *HaP110* were knocked down effectively at the protein and mRNA levels (S6 Fig). These results suggested that HaP60 is necessary for the IIS cascade and ultimate cell proliferation.

### HaP60 promotes cell apoptosis under 20E regulation

**Fig 6:**
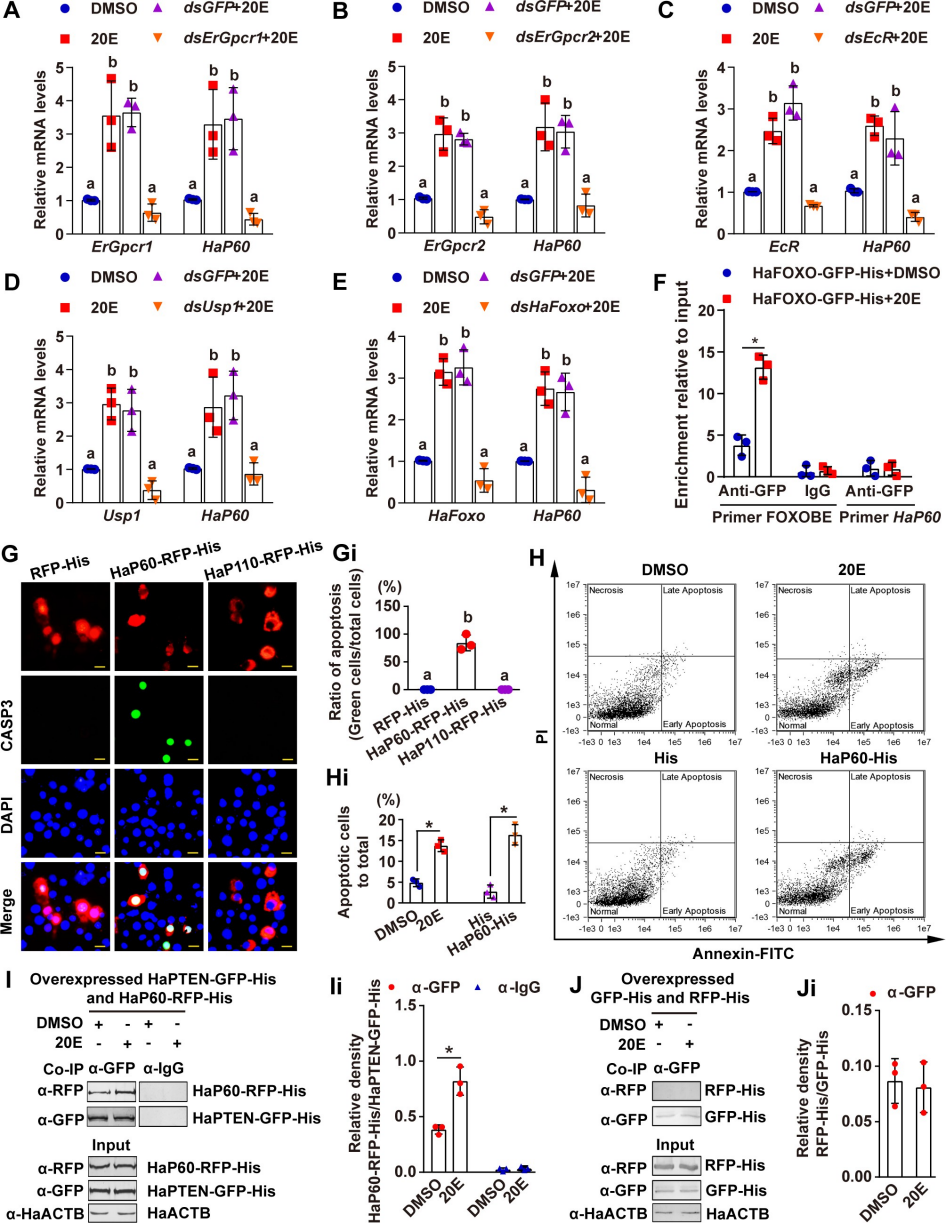
20E induced HaP60 expression via HaFOXO and interaction with HaPTEN to promote apoptosis. **A-E.** *ErGpcr1*, *ErGpcr2*, *EcR*, *Usp1*, and *HaFoxo* knockdown decreased *HaP60* expression under 20E induction. **F.** Ch-IP assay showing 20E promoted *HaP60* expression via HaFOXO binding to FOXOBE and detected by qRT-PCR. IgG is a negative control. Primer FOXOBE targeting FOXOBE. Primer HaP60, as non-FOXOBE control targeting to HaP60 open reading frame (ORF). **G.** CASP3 activity in RFP-His, HaP60-RFP-His, and HaP110-RFP-His overexpressing HaEpi cells. Red: RFP-His, HaP60-RFP-His, and HaP110-RFP-His; green: CAPS3 activity; blue: nuclei stained by DAPI. Bar, 20 μm. **Gi.** Statistical analysis for G. **H.** Flow cytometry showing the role of HaP60 in apoptosis. **Hi.** Statistical analysis for H. **I.** 20E induced the HaP60–HaPTEN interaction. HaP60-RFP-His and HaPTEN-GFP-His were overexpressed together in HaEpi cells. 20E (5 μM, for 6 h), DMSO as solvent control. HaPTEN-GFP-His was immunoprecipitated with anti-GFP antibody (α-GFP) and the coprecipitated HaP60-RFP-His was examined by western blotting using anti-RFP antibody (α-RFP). Mouse IgG (α-IgG) was used as a negative control for the antibody. **Ii.** Statistical analysis for I. **J and Ji.** RFP-His and GFP-His tag control for I. *P < 0.05 using two-tailed Student’s *t*-test for two pairs analysis, or different lowercase letters indicate significant differences (*P* < 0.05) using one-way ANOVA for multiple analysis.

The signaling axis of 20E upregulation of *HaP60* transcription was addressed in HaEpi cells. 20E induced *HaP60* expression; however, this activity was repressed after knockdown of *ErGpcr1*, *ErGpcr2*, *EcR*, *Usp1*, and *HaFoxo* (Fig 6A–6E), suggesting that 20E acts via the ErGPCRs-, EcR- and HaFOXO- signaling axis to upregulate *HaP60* expression. A FOXO binding element (FOXOBE) was further identified in the promoter of *HaP60* 5′-_-863_TTGTTGTC_-856_-3′, which was similar to the typical FOXOBE 5′-TTGTTTAC-3′ [26]. A chromatin immunoprecipitation (ChIP) assay showed that 20E increased *HaP60* transcription by HaFOXO binding to FOXOBE (Fig 6F). Thus, 20E acts via HaFOXO to upregulate *HaP60* expression.

Given that HaP60 was upregulated in the metamorphic stages by 20E, we characterized the role of HaP60 in apoptosis by overexpressing HaP60-red fluorescent protein (RFP)-His and HaP110-RFP-His in HaEpi cells, with RFP-His as a tag control. Higher CASP3 activity was detected in the HaP60-RFP-His overexpressing cells; however, less CASP3 signal was detected in the RFP-His and HaP110-RFP-His overexpressing cells (Fig 6G and 6Gi), when RFP-His, HaP60-RFP-His, and HaP110-RFP-His were expressed equally (S7 Fig). Flow cytometry analysis demonstrated that HaP60-His increased the number of apoptotic cells (Fig 6H and 6Hi). The P85α-PTEN complex causes G2/M phase arrest and apoptosis [27]; therefore, HaPTEN-GFP-His and HaP60-RFP-His were overexpressed in HaEpi cells to detect the interaction between HaPTEN and HaP60, and to identify the mechanism by which HaP60 induces apoptosis. The results showed the HaPTEN-GFP-His and HaP60-RFP-His were immunoprecipitated using anti-GFP antibodies in DMSO treatment, and 20E increased the amount of HaP60-RFP-His, compared with that induced by DMSO (Fig 6I and 6Ii). RFP and GFP did not interact in these experiments (Fig 6J and 6Ji). These data confirmed that HaP60 promotes apoptosis by interacting with HaPTEN under 20E regulation.

### Both HaP60 and HaP110 are necessary for larval growth and metamorphosis

**Fig 7:**
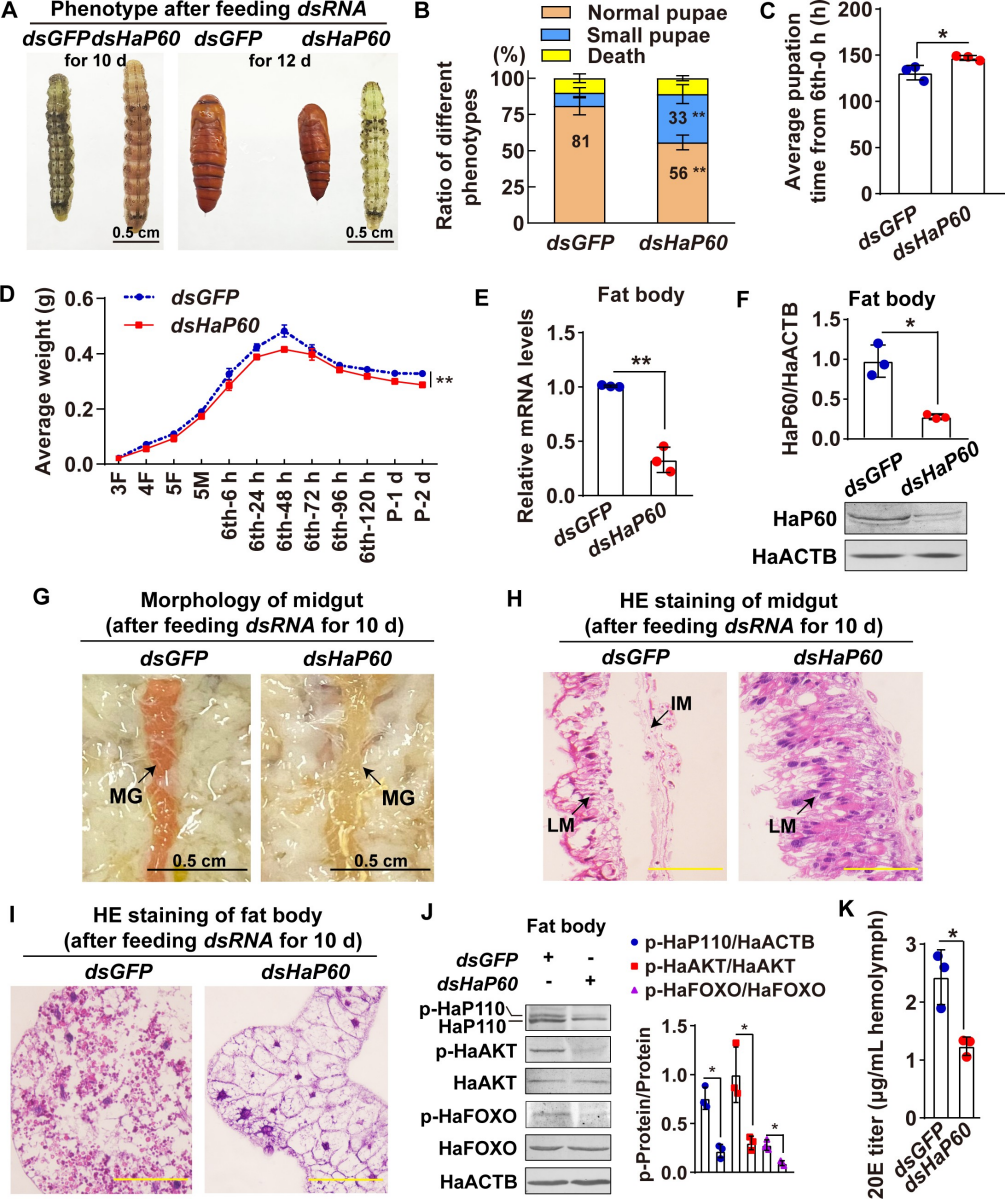
Larvae fed with *E*. *coli* HT115 that expressed *dsHaP60* led to small pupa and delayed pupation. **A.** Phenotype after feeding *dsHaP60* for 10 d and 12 d. Bar, 0.5 cm. **B.** Statistical analysis of the phenotype in A using Student’s *t*-test based on three repeats. Thirty larvae for each repeat. **C.** The pupation time from 6th-6 h to pupa 0 d. **D.** The average body weight statistics of insects from the third instar feeding (3F) larvae to pupae 2 days (P-2 d) by Student’s *t*-test based on three repeats. Thirty larvae for each repeat. **E and F.** Efficiency analysis of *HaP60* knockdown using qRT-PCR and western blotting at the mRNA and protein levels. **G.** Morphology of midgut after feeding *dsRNA* for 10 d. MG: midgut. Bar, 0.5 cm. **H and I.** HE staining of midgut and fat body after feeding *dsRNA* for 10 d. LM: larval midgut; IM: imaginal midgut. Bars, 20 μm. **J.** *HaP60* knockdown decreased HaAKT and HaFOXO phosphorylation after feeding *dsRNA* for 8 d. **K.** 20E titer detection in the hemolymph after feeding *dsRNA* for 10 d. *P < 0.05 and **P < 0.01 using two-tailed Student’s *t*-test. The bars indicate the mean ± SD.

To determine the role of HaP60 in larval growth and metamorphosis, we knocked down *HaP60* by feeding third instar larvae with *E*. *coli* HT115 that expressed *dsGFP* or *dsHaP60*, respectively. Phenotype analysis showed the larvae produced 33% small pupae after feeding with *dsHaP60*, which was significantly higher than the 9% small pupae induced by feeding with *dsGFP* (Fig 7A and 7B). Meanwhile, pupation time was delayed for 16 h, and the average weight of the pupa was decreased, compared with those in larvae fed with *dsGFP* (Fig 7C and 7D) when *HaP60* was confirmed as being knocked down according to its mRNA and protein levels (Fig 7E and 7F). The midgut appeared red at 10 d post-feeding *dsGFP*, whereas, the larval midgut was not red and did not separate from the imaginal midgut at the same time after feeding with *dsHaP60* (Fig 7G and 7H). Moreover, the larval fat body retained complete after feeding with *dsHaP60* at 10 d, whereas the larval fat body started to decompose in the *dsGFP*-fed control (Fig 7I). Western blotting analysis showed that HaP110, HaAKT, and HaFOXO phosphorylation decreased after *HaP60* knockdown (Fig 7J). Furthermore, the 20E titer was decreased at 10 d after *HaP60* knockdown (Fig 7K). These results suggested that HaP60 is necessary for larval growth and 20E production, and apoptosis of larval tissues during metamorphosis.

**Fig 8:**
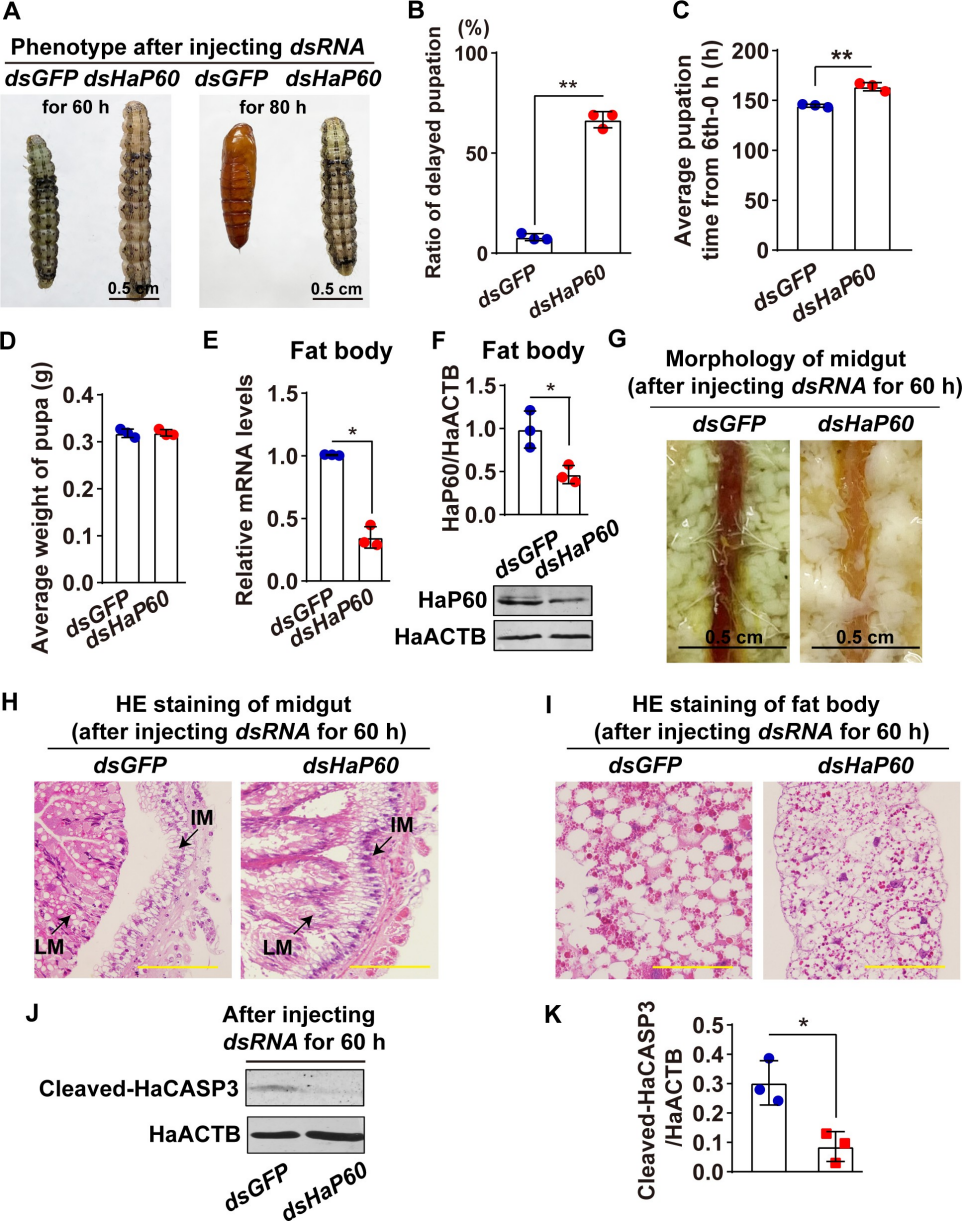
HaP60 knockdown by injecting *dsRNA* at the wandering stages delayed metamorphosis. **A.** Phenotype analysis after first injecting *dsRNA* for 60 h and 80 h. Bar, 0.5 cm. **B.** Statistical analysis of the delayed pupation in A by Student’s *t*-test based on three repeats. Thirty larvae for each repeat. **C.** Statistical analysis of pupation time from 6th-0 h to pupa 0 d after *HaP60* knockdown. **D.** The average weight of pupa after *HaP60* knockdown. Thirty larvae for each repeat. **E and F.** Efficiency analysis of *HaP60* knockdown using qRT-PCR and western blotting at the mRNA and protein levels. **G.** Morphology of midgut after first injecting *dsRNA* for 60 h. Bar, 0.5 cm. **H and I.** HE staining of midgut and fat body after first injecting *dsRNA* for 60 h. LM: larval midgut; IM: imaginal midgut. Bars, 20 μm. **J and K.** The western blotting analysis of cleaved-HaCASP3 after first injecting *dsRNA* for 60 h. *P < 0.05 and **P < 0.01 using two-tailed Student’s *t*-test. The bars indicate the mean ± SD.

To address the role of the dephosphorylation of HaP60 in the metamorphosis, *dsHaP60* was injected into the 6th-72 h larva that HaP60 was dephosphorylated to knock down *HaP60*, *dsGFP* as control. Phenotype analysis showed *HaP60* knockdown caused 67% delayed pupation, which was dramatically higher than 8% *dsGFP* (Fig 8A and 8B), and the pupation time was delayed approximately for 19 h after *HaP60* knockdown (Fig 8C), and the pupa weight was no difference between *dsHaP60* and *dsGFP* when *HaP60* was knocked down in the mRNA and protein levels (Fig 8D–8F). Meanwhile, the PCD of the midgut and fat body was also delayed after *HaP60* knockdown (Fig 8G–8I). Furthermore, cleaved-HaCASP3, an activated form of HaCASP3 and a marker of PCD, was reduced after *HaP60* knockdown (Fig 8J and 8K), suggesting non-phosphorylated HaP60 is necessary for apoptosis of larval tissue and metamorphosis.

**Fig 9:**
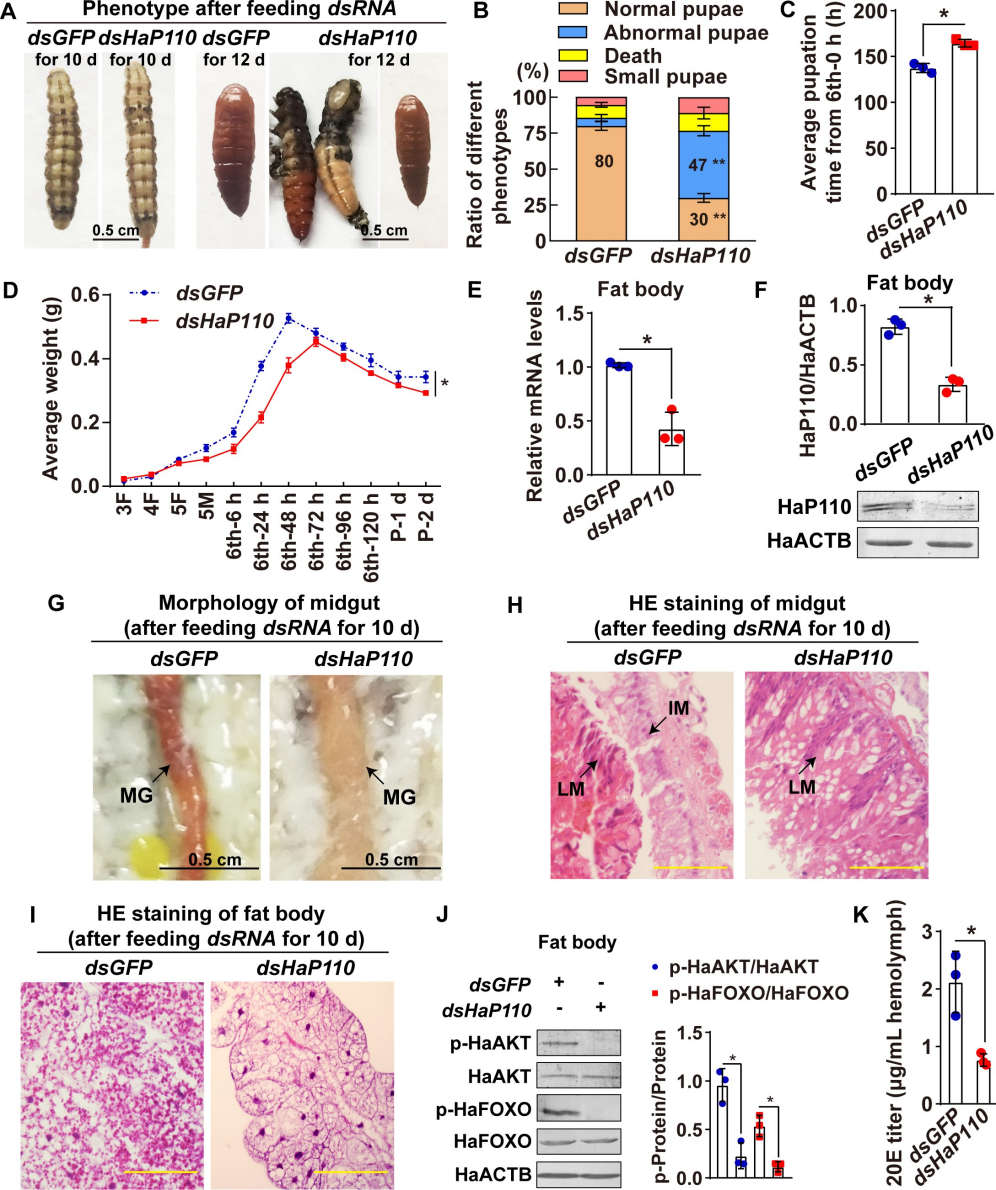
Larvae fed with *E*. *coli* HT115 that expressed *dsHaP110* resulted in abnormal pupa and delayed pupation. **A.** Phenotype after feeding *dsHaP110* for 10 d and 12 d. Bar, 0.5 cm. **B.** Statistical analysis of the phenotype in A by Student’s *t*-test based on three repeats. Thirty larvae for each repeat. **C.** The pupation time from 6th-6 h to pupa 0 d. **D.** The average body weight statistics of insects from 3F larvae to P-2 d pupae by Student’s *t*-test based on three repeats. Thirty larvae for each repeat. **E and F.** Efficiency analysis of *HaP110* knockdown using qRT-PCR and western blotting at the mRNA and protein levels. **G.** Morphology of midgut after feeding *dsRNA* for 10 d. MG: midgut. Bar, 0.5 cm. **H and I.** HE staining of midgut and fat body after feeding *dsRNA* for 10 d. LM: larval midgut; IM: imaginal midgut. Bars, 20 μm. **J.** *HaP110* knockdown decreased HaAKT and HaFOXO phosphorylation after feeding *dsRNA* for 8 d. **K.** 20E titer detection in hemolymph after feeding *dsRNA* for 10 d. *P < 0.05 using two-tailed Student’s *t*-test. The bars indicate mean ± SD.

*HaP110* knockdown caused 47% abnormal pupae, which was higher than the 6% abnormal pupation induced by feeding with *dsGFP* (Fig 9A and 9B), suggesting a different role of the catalytic subunit HaP110 from that of the regulatory subunit HaP60. Other effects were similar to those induced by *HaP60* knockdown, including delayed pupation time for 27 h (Fig 9C) and decreased pupa weight (Fig 9D) when *HaP110* was knocked down at the mRNA and protein levels in the fat body (Fig 9E and 9F). The PCD of the larval midgut and fat body was also delayed after feeding *dsHaP110*, compared with *dsGFP* feeding (Fig 9G–9I). Western blotting analysis showed that HaAKT and HaFOXO phosphorylation decreased after *HaP110* knockdown (Fig 9J). Moreover, the 20E titer was reduced at 10 d after *HaP110* knockdown (Fig 9K). These results revealed that HaP110 promotes larval growth and 20E production to initiate metamorphosis.

## Discussion

PI3Ks play pivotal roles in insulin and other cell proliferation pathways via the incorporation of subunits P110 and P85/P60. Steroid hormones, including 20E, counteract ILPs activity; however, the mechanism by which 20E regulates PI3K is poorly understood. The present study determined that HaP60 plays dual roles in cell proliferation and apoptosis in the IIS and 20E pathways. ILPs induce phosphorylation of HaP60 and HaP110; however, 20E induces dephosphorylation of HaP60 and HaP110 via HaPTPN6 to block the IIS, which ceases HaFOXO to localize to the nucleus. 20E upregulates HaP60 expression via HaFOXO, which interacts with HaPTEN to induce apoptosis.

### HaP60 functions in cell proliferation and apoptosis by changing its phosphorylation status

There are many isoforms of PI3K in mammals. For example, class IA PI3Ks consist of P110 catalytic subunits (P110α, P110β, and P110δ, encoded by genes *PIK3CA*, *PIK3CB*, and *PIK3CD*, respectively) and P85 regulatory subunits (P85α, P85β, and P55γ, encoded by *PIK3R1*, *PIK3R2*, and *PIK3R3*, respectively). Other regulatory isoforms of P50α and P55α are generated by splicing of P85α transcripts [2]. Five mammalian regulatory subunit isoforms of class IA PI3Ks can act as positive and negative regulators of PI3K activity in different cell types and response to different stimuli [2]. Each regulatory subunit of PI3K is essential for the cell membrane translocation and phosphorylation of P110, the subsequent AKT and FOXO phosphorylation, and the cytosolic localization of FOXO, finally resulting in cell proliferation [6]. However, there is also a report that P85α reduces PI3K activity and insulin signaling [28]. P50α/P55α represses insulin sensitivity [29] and P55γ suppresses vascular smooth muscle cell proliferation [11]. These contradictory roles of P85/P60 to promote cell proliferation and apoptosis are confusing.

P60 and P110 are unique forms of class IA PI3K in *D*. *melanogaster* [30], which presents a simple model compared with the complicated composition of PI3Ks in mammals to address the regulatory mechanism of P85/P60 functions. Insect larval growth is regulated mainly by ILPs and metamorphosis is regulated by 20E [31]. The IIS is conserved from insects to humans [15]. 20E, a steroid hormone ecdysone produced in insects, is known to oppose ILPs function at high levels [32]. According to the variation of HaP60 phosphorylation and expression levels during *H*. *armigera* development from larval growth to metamorphosis, we revealed that HaP60 promotes cell proliferation as a phosphorylated form under ILPs regulation for larval growth, but HaP60 promotes apoptosis as a dephosphorylated form under 20E regulation for metamorphosis. This finding explains the mechanism by which HaP60 promotes both cell proliferation and apoptosis.

### 20E upregulates HaPTPN6 to dephosphorylate HaP60 and HaP110 to block IIS and stop larval growth

How animals stop the growth and maintain their body size has been studied for a long time and has remained poorly understood until now. The known mechanisms include the coordination of ILPs and steroid hormones to determine body size in insects [33]. The PGs grow as the body grows to produce more 20E [34]. The 20E titer greatly increases during metamorphosis and competes for dopamine to bind the receptor, DopEcR, to stop feeding behavior [35]. 20E suppresses dMyc activity, which is involved in IIS and TOR (the target of rapamycin) pathway, and global growth at the larval-pupal transition [36]. Moreover, 20E signaling increases Imaginal morphogenesis protein-Late 2 (Imp-L2), a *Drosophila* homolog of insulin-like growth factor-binding protein 7 (IGFBP7), which circulates in the hemolymph to bind and antagonize ILP, thereby attenuating peripheral IIS and body growth [37]. Furthermore, 20E reduces microRNA miR-8 expression, which activates PI3K, thereby facilitating fat cell proliferation and organism growth, whereas 20E upregulates u-shaped (USH), a target of miR-8, and inhibits PI3K activity and blocks cell growth [38, 39]. However, the mechanism of the regulation of 20E on post-translational modification of PI3K to antagonize IIS is unclear. PTPN6 is reported to show high expression in insulin target tissues in obesity, in which it interacts with P85, resulting in P85 dephosphorylation and reduced AKT phosphorylation in the control of liver metabolism [40, 41]; however, the mechanism is unclear. In the present study, we further revealed that 20E upregulates HaPTPN6 expression via the ErGPCRs- and EcR- signaling axis, which interacts with HaP60 and results in HaP60 dephosphorylation in *H*. *armigera*. The dephosphorylation of HaP60 blocks the IIS from the upstream part of the pathway, thus repressing the subsequent phosphorylation of HaP110, HaAKT, and HaFOXO, which finally results in HaFOXO entering the nucleus to initiate gene transcription for apoptosis. Our study revealed that steroid hormone 20E upregulation of HaPTPN6 expression to dephosphorylate HaP60 counteracts the IIS and is one of the mechanisms by which animals stop growing.

### 20E promotes HaP60 expression and utilizes HaP60 to induce apoptosis

Overexpression of P60 and a P60 mutant that lacked the inter-SH2 domain for binding to P110, caused growth arrest in the first instar for as long as 2 weeks in *D*. *melanogaster*, equivalent to a phenotype of PTEN overexpression [42]; however, the mechanism was unclear. 20E promotes HaPTEN expression, which reverses PI3K activity, and results in cell apoptosis in *H*. *armigera* [43]. Our research further demonstrated that 20E, via ErGPCRs-, EcR- and HaFOXO-signaling promotes HaP60 expression directly by HaFOXO binding to FOXOBE in the *HaP60* promoter. The highly expressed HaP60 interacts with HaPTEN under 20E mediation, which further represses the IIS and results in HaFOXO localizing to the nucleus to induce apoptosis.

**Fig 10:**
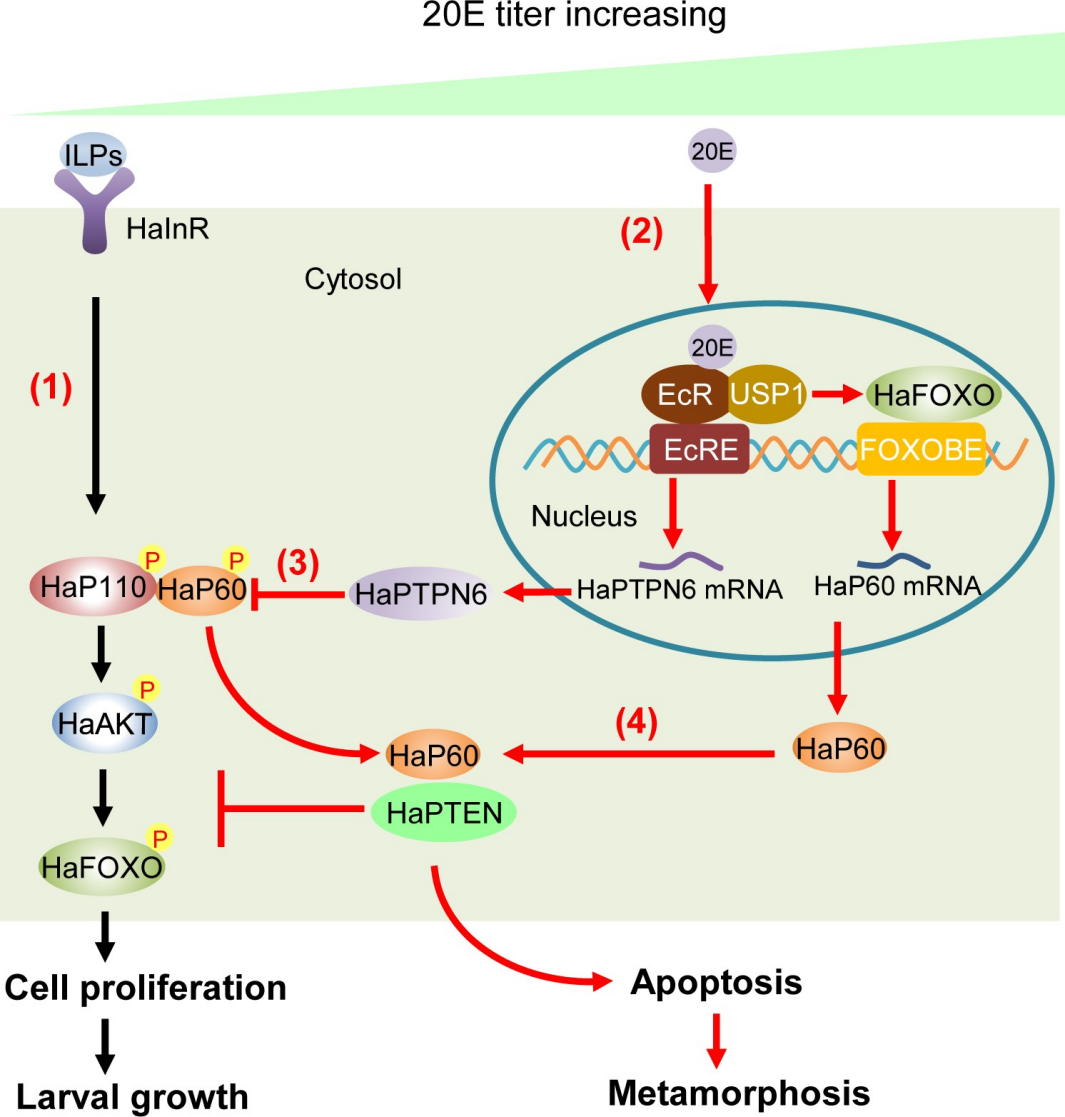
Chart interpreting the dual functions of HaP60 in the IIS and 20E pathways by changing its phosphorylation status and the consequence. **(1).** HaP60 plays a key role in the IIS as a phosphorylated form to mediate translocation of HaP110 to the cell membrane and HaAKT and HaFOXO phosphorylation to arrest HaFOXO in the cytosol to allow cell proliferation and larval growth. **(2 and 3).** 20E, via the EcR, upregulates HaPTPN6 expression to dephosphorylate HaP60 and HaP110, therefore blocking IIS. **(4).** 20E, via the EcR-and HaFOXO- signal axis, upregulates HaP60 expression and mediates the interaction of HaP60 with HaPTEN to further reverse the function of HaP110, resulting in cell apoptosis and metamorphosis.

Our results demonstrated that HaP60 plays dual roles in the IIS and 20E pathways. HaP60 is necessary for HaP110 phosphorylation and cell membrane translocation, and the subsequent cascade of protein phosphorylation, resulting in cell proliferation. 20E promotes HaP60 and HaP110 dephosphorylation by upregulating HaPTPN6 expression, thereby blocking cell membrane translocation and decreasing the HaP60 and HaP110 interaction. 20E, via the ErGPCRs-, EcR-, and HaFOXO- signal axis, promotes the expression of HaP60, which interacts with HaPTEN to reverse HaP110 action and bring about cell apoptosis at the metamorphic stages (Fig 10).

## Materials and methods

### Insects

*Helicoverpa armigera* were cultivated in an insect laboratory with a stable temperature of 27°C and a 14 h light/dark 10 h cycle. The larvae were fed with artificial feed according to a previous study [44].

### Cell culture

The *H*. *armigera* epidermis (HaEpi) cell line was developed from the *H*. *armigera* epidermis and cultured in Grace’s medium (11300–043, Gibco, Carlsbad, CA, USA) with 10% fetal bovine serum (FBS; 04-001-1ACS, BioInd, Kibbutz Beit Hawmek, Israel) at 27°C.

### Preparation of HaP60 and HaP110 antibodies

Partial fragments of *HaP60* or *HaP110* were amplified using primers shown in S1 Table and fused with pET-30(a) to construct the expression plasmid. Genbank numbers of genes were shown in S2 Table. The plasmid was then transformed into the *Escherichia coli* rosetta strain grown for 3 h at 37°C. Expression was then induced by adding 0.5 mM isopropyl β-D-Thiogalactoside (IPTG; A600168, Sangon Biotech, Shanghai, China) overnight at 25°C. The bacterial cells were collected, suspended with phosphate-buffer saline (PBS), broken using ultrasound, centrifuged at 10000 × *g* for 20 min, and the pellet was retained. The protein was denatured using 8 M urea, pH 8.0, purified using a His-tag column, and renatured by reducing the urea concentration step by step in the dialysate. The protein was then mixed with adjuvant and injected into a rabbit to produced anti-HaP60 antibodies or anti-HaP110 antibodies, according to a previously described method [45].

### The antibodies used in this study

Monoclonal anti-Actin Beta (ACTB) antibody (AC026, ABclonal Technology, Wuhan, China), monoclonal anti-GFP antibody (AE012, ABclonal Technology), monoclonal anti-RFP antibody (AE020, ABclonal Technology), monoclonal anti-His antibody (AE003, ABclonal Technology), polyclonal anti-phospho-AKT antibodies (AP0140, ABclonal Technology), polyclonal anti-phospho-FOXO antibodies (AP0684, ABclonal Technology), mouse control IgG (AC011, ABclonal Technology), rabbit control IgG (AC005, ABclonal Technology), Horseradish Peroxidase (HRP)-conjugated goat anti-rabbit IgG light chain (AS061, ABclonal Technology), polyclonal anti-AKT antibodies (WL0003b, Wanleibio, Shenyang, China), polyclonal anti-FOXO antibodies (WL02891, Wanleibio), Alkaline phosphatase (AP)-conjugated horse anti-mouse IgG (ZB2310, ZSGB-BIO, Beijing, China), and AP-conjugated goat anti-rabbit IgG (ZB2308, ZSGB-BIO).

### Lambda protein phosphatase (λPPase) treatment

The epidermis, midgut, and fat body of the 6th-48 h and 6th-96 h larvae were extracted using lysate buffer (P0013J, Beyotime Biotechnology, Shanghai, China) with 1 mM phenylmethanesulfonyl fluoride (PMSF; A100754, Sangon Biotech), followed by centrifugation at 10000 × *g* at 4°C for 10 min. The supernatant was incubated with λPPase according to the kit’s instruction (P0753L, New England Biolabs, Ipswich, MA, USA), and analyzed using western blotting.

### Western blotting

Western blotting analysis was conducted according to our previous method [46]. Epidermis, midgut, and fat body were extracted using 40 mM Tris-HCl, pH 7.5, with 1 mM PMSF, followed by centrifugation at 15000 × *g* at 4°C for 15 min. The supernatant was obtained and the protein content was determined using Bradford’s method. Proteins (50 μg) were separated using SDS-PAGE (10% SDS-PAGE for HaP60 and other proteins detection, 6% SDS-PAGE for HaP110 detection) and transferred onto nitrocellulose membranes. Non-specific binding was blocked using 5% non-fat milk in TBST (Tris-buffered saline tween; 150 mM NaCl, 10 mM Tris-HCl, 0.05% Tween-20, pH 7.5) for 1 h and then the membranes were incubated with anti-HaP60 antibodies (diluted in a ratio of 1:100) or anti-HaP110 antibodies (diluted in a ratio of 1:100) overnight at 4°C. Then, the membranes were washed with TBST three times for 5 min each and incubated with the secondary antibody (AP-conjugated goat anti-rabbit IgG, 1:5000 in the blocking buffer) using our previously reported method. HaACTB, as a protein quality control, was detected using anti-HaACTB antibodies. Anti-GFP, anti-RFP, and anti-His (1:2000 in the blocking buffer) antibody were used to detect the GFP tag, RFP tag, and His tag protein, together with the same secondary antibody (AP-conjugated horse anti-mouse IgG, 1:5000 in the blocking buffer).

### Immunohistochemistry

The tissues were fixed in 4% paraformaldehyde (PFA) and submitted to a professional company (Servicebio, Wuhan, China). Anti-HaP60 antibodies (diluted in 1:50) and anti-HaP110 antibodies (diluted in 1:50) were used to detect the locations of HaP60 and HaP110 in the midgut, and the secondary antibody, fluorescein isothiocyanate (FITC)-conjugated goat anti-rabbit IgG (H+L) (1:200; GB22303, Servicebio) was used to show the signal. Fluorescence was observed under an Olympus BX51 fluorescence microscope (Olympus Optical Co, Tokyo, Japan).

### Hormonal treatment in the larvae

Different concentrations (1, 2, 5, and 10 μg/larva) of human insulin (P3376, Beyotime Biotechnology) or 20E (50, 100, 200, and 500 ng/larva; H5142, Sigma- Aldrich, St. Louis, MO, USA) diluted with PBS and then 5 μL of insulin or 20E were injected into the hemolymph of the 6th-6 h larva for 6 h, individually. Equally diluted PBS for insulin and DMSO for 20E were used as controls. Insulin at 5 μg/larva or 20E at 500 ng/larva was injected into the hemolymph of the 6th-6 h larva from 1 h to 24 h; PBS and DMSO were used as controls. Each treatment used three larvae, from which the fat body was extracted using 40 mM Tris-HCl for western blotting.

### Quantitative real-time reverse transcription PCR (qRT-PCR)

Total RNA was extracted using TransZol (ET101-01, TransGen Biotech, Beijing, China), and the first-strand cDNA was synthesized according to the manufacturer’s instructions (G492, Abcam, Cambridge, UK). Then, qRT-PCR was performed with a CFX96 real-time system (Bio-Rad, Hercules, CA, USA) using 2 × SYBR qRT-PCR pre-mixture (PC3301, Aidlab, Beijing, China). The relative mRNA expression level was calculated by R = 2^−ΔΔCT^ method (ΔΔCT = ΔCT_sample_-ΔCT_control_, ΔCT_control_ = CT_*gene*_-CT_*HaActb*_), with *HaActb* as the control.

### Co-immunoprecipitation (Co-IP)

For the HaPTEN and HaP60 Co-IP assay, 4 μg HaPTEN-GFP-His and HaP60-RFP-His plasmids were co-transfected into HaEpi cells for 72 h using the Quick Shuttle-enhanced transfection reagent (KX0110042, Biodragon Immunotechnologies, Beijing, China), followed by 20E treatment for 6 h, with DMSO as the control. Anti-GFP antibodies (1:100) were used to immunoprecipitate HaPTEN-GFP-His. Then, the cells were lysed using lysate buffer (P0013, Beyotime Biotechnology) containing phosphatase inhibitor and protease inhibitor, and the supernatant was collected by centrifugation at 10000 × *g* for 15 min at 4°C. The supernatant was added with 50 μL of protein A (SA012005, Smart-Lifesciences, Changzhou, China), and shaken at 4°C for 1 h to remove non-specific binding. Cell lysate (20 μL) was extracted as input. The antibody was added into cell lysate overnight at 4°C, and then 50 μL of protein A was added to the cell lysate, incubated for 4 h, and then washed with lysate buffer. Thereafter, 40 μL of lysate buffer was used to resuspend the protein A, which was then analyzed using western blotting. For the HaPTPN6 and HaP60 Co-IP assay, 4 μg HaPTPN6-GFP-His plasmid was transfected into HaEpi cells for 72 h, which were then treated with 5 μg/mL insulin for 1 h and 5 μM 20E for 6 h or only treated with 5 μM 20E for 6 h, DMSO as control, and CNBr-activated Sepharose 4B conjugated with anti-GFP antibody to immunoprecipitate HaPTPN6-GFP-His. For HaP60 and HaP110 Co-IP assay, cells were added with 5 μg/mL insulin for 1 h, followed by 5 μM 20E for 6 h. Then, the cells were lysed using a lysate buffer containing phosphatase inhibitor and protease inhibitor, and the supernatant was collected by centrifugation 10000 × *g* for 15 min at 4°C and incubated with CNBr-activated Sepharose 4B conjugated with anti-HaP60 antibodies overnight at 4°C to immunoprecipitate HaP60. The CNBr beads were washed with wash buffer (0.1 M Tris-HCl, 0.1 M NaCl, pH 8.0), and the complex conjugated by CNBr beads were eluted using buffer (0.1 M glycine, pH 2.5) followed by neutralization, before being subjected to western blotting.

### Inhibitor assay

HaEpi cells were treated with 1% phosphatase inhibitor (PPI) (M7528, AbMole BioScience, Houston, TX, USA) or 50 μg/mL Cycloheximide (CHX) (HY-12320, MedChemExpress, Monmouth Junction, NJ, USA), followed by 5 μg/mL insulin for 1 h, and then treated with 5 μM 20E for 6 h, before being analyzed using western blotting. Different concentrations TPI-1 (0, 2, 5, 10, 20 ng/larva; abs812922, Absin, Shanghai, China) were injected into 6th-60 h larva for 12 h, and then the fat body proteins were extracted and analyzed using western blotting. In HaEpi cells, different concentrations TPI-1 (50, 100 nM) and 10 μM BN82002 (HY-112776, MedChemExpress) were added respectively, followed by treatment with 5 μg/mL insulin for 1 h and with 5 μM 20E for 6 h, and then analyzed using western blotting.

### Immunocytochemistry and phospho-histone H3 detection

For HaP60 and HaP110 subcellular translocation, the cells were treated with 100 nM TPI-1, and then 5 μg/mL insulin for 1 h, followed by 5 μM 20E for 6 h. To investigate HaP60’s function in HaP110 subcellular translocation, cells were transfected with 2 μg *dsHaP60* (*dsRNA* targeting *HaP60*) or *dsGFP* (*dsRNA* targeting green fluorescent protein gene), respectively, and then treated with insulin for 1 h. Cell proliferation was detected by observing phospho-histone H3 using anti-phospho-histone H3 antibodies (Ser10) (9701, Cell signaling technology, Danvers, MA, USA), in which the cells were transfected with 2 μg *dsHaP60*, *dsHaP110* (*dsRNA* targeting *HaP110*), and *dsGFP*, respectively, and treated with insulin for 1 h. Then, the cells were washed with PBS, fixed in 4% PFA, and blocked using 5% bovine serum albumin (BSA; A8020, Solarbio Life Sciences, Beijing, China). The cells were then incubated with anti-HaP60, anti-HaP110 antibodies (1:50), or anti-phospho-histone H3 antibodies (Ser10) (1:800) overnight at 4°C, before being washed with PBS. The cells were then incubated with goat anti-rabbit IgG Alexa Fluor 488 (R37116, Thermo Fisher Scientific, Waltham, MA, USA) (1:1000 diluted in blocking buffer) for 1 h at 37°C, and washed with PBS. The cell membrane was stained using Fluor 594-conjugated wheat germ agglutinin (WGA) (W21404, Thermo Fisher Scientific) for 5 min, and washed with PBS. The cell nuclei were stained using 10 μg/mL 4’,6-diamidino-2-phenylindole (DAPI) (KGA215, KeyGEN Biotechnology, Nanjing, China) for 10 min and washed with PBS. The cells were observed under an Olympus BX51 fluorescence microscope (Shinjuku-ku, Tokyo, Japan).

### CASP3 activity detection

HaEpi cells were transfected with 4 μg RFP-His, HaP60-RFP-His, or HaP110-RFP-His for 72 h, respectively, or treated with 5 μM 20E for 72 h, and then incubated with 2 μM NucView 488 caspase-3 assay kit (30029, Biotium, Fremont, CA, USA) for 30 min at room temperature. The cells were then fixed in 4% PFA, washed with PBS, and the nuclei were stained with DAPI for 10 min. Finally, the CASP3 signal was observed under an Olympus BX51 fluorescence microscope.

### RNA interference in the HaEpi cell line

Cells were transfected with 2 μg *dsErGpcr1* (*dsRNA* targeting *ErGpcr1*), *dsErGpcr2* (*dsRNA* targeting *ErGpcr2*), *dsEcR* (*dsRNA* targeting *EcR*), *dsUsp1* (*dsRNA* targeting *Usp1*), and *dsHaFoxo* (*dsRNA* targeting *HaFoxo*), respectively, into HaEpi cells for 48 h, *dsGFP* was used as a control. All transfected cells were treated with 5 μM 20E for 6 h, with DMSO as a control. For the *HaPtpn6* knockdown assay, 2 μg *dsHaPtpn6* (*dsRNA* targeting *HaPtpn6*) was transfected into HaEpi cells, which were then incubated with 5 μg/mL insulin for 1 h, followed 5 μM 20E for 6 h, with *dsGFP* as control. The total RNA or protein was extracted and analyzed by qRT-PCR or western blotting, respectively.

### Chromatin immunoprecipitation (ChIP) assay

For EcRE detection, 4 μg EcR-RFP-His were transfected into HaEpi cells for 72 h, which were then treated with 5 μM 20E for 6 h. For FOXOBE detection, 4 μg HaFOXO-GFP-His were transfected into HaEpi cells for 72 h, which were then treated with 5 μM 20E for 6 h. The cells were treated according to the instruction of the ChIP assay kit (P2078, Beyotime Biotechnology). Anti-His antibody was used to detect EcR-RFP-His, anti-GFP antibody was used to detect HaFOXO-GFP-His, and IgG was used as a negative control.

### Flow cytometry detection of apoptosis

Apoptosis was detected using flow cytometry with an Annexin-FITC apoptosis detection kit (C1062M, Beyotime Biotech). HaEpi cells were transfected with 4 μg HaP60-His and His respectively for 72 h or treated with 5 μM 20E for 72 h, with DMSO as a control for 20E. The cells were washed with PBS, lysed with 0.5% trypsin, and collected by centrifugation at 1000 × *g* for 5 min at 4°C and then treated according to Annexin-FITC apoptosis detection kit to detect apoptosis by flow cytometry.

### *E*. *coli* HT115-expressed *dsRNA* feeding to larvae

Gene fragments of *dsHaP60*, *dsHaP110*, *dsHaPtpn6*, and *dsGFP* were amplified, fused with vector L4440, provided by Dr. Marek Jindra (Biology Center ASCR, Czech Republic), and then transformed into *E*. *coli* DH5α. The constructed plasmids were purified and then transferred into *E*. *coli* HT115, provided by Dr. Marek Jindra. HT115 cells expressing *dsHaP60*, *dsHaP110*, *dsHaPtpn6*, and *dsGFP* were inoculated into 300 mL of medium (pH 7.4), shaken for 3 h at 37°C, and then induced using IPTG for 5 h. Subsequently, the bacterial culture was centrifuged at 10000 × *g* for 5 min at 4°C. The bacteria were resuspended in 500 μL PBS, pH 7.4. PBS with bacteria expressed *dsHaP60*, *dsHaP110* or *dsHaPtpn6* was applied to the surface of forage that was fed to larva every day up to the 6th-72 h stage. Bacteria expressing *dsGFP* were fed at an equal amount at the same time. For *HaPtpn6* knockdown in the larva, 2 μg *dsHaPtpn6* were injected into the 6th-72 h larva after feeding *E*.*coli* expressing *dsHaPtpn6* at the last day for 24 h, and the second injection was performed at the 6th-96 h, and then the last injection was performed at the 6th-120 h. The interference efficiency was checked using qRT-PCR and western blotting.

### RNAi knockdown by injecting *dsRNA* into larva

For *HaP60* knockdown in the larva, 2 μg *dsHaP60* were injected into the 6th-72 h larva for 24 h, *dsGFP* as a control. The second injection was conducted at the 6th-96 h, and then the last injection was performed at the 6th-120 h to observe phenotype and analyze the pupation time and pupal weight. The total RNA or protein was extracted after the second injection for 12 h to detect the RNAi efficiency of *HaP60* knockdown.

### 20E titer detection

Hemolymph (100 μL) from more than three larvae was frozen in the liquid nitrogen overnight and freeze-dried for 6 h. The samples were then ground in 500 μL of 80% methanol and centrifuged at 10000 × *g* at 4°C for 10 min. The supernatant was air-dried at room temperature for 3 h and dissolved in 100 μL of enzyme immunoassay (EIA) buffer to detect the 20E titer using a 20E Enzyme Immunoassay kit (A05120, Bertin Pharma, Montignyle-Bretonneux, France).

### Phylogenetic analysis

The phylogenetic analysis was conducted requiring four steps by MEGA 5.0, a program for phylogenetic analysis in a single environment. We identified and acquired *Homo sapiens*, *Drosophila melanogaster*, *Bombyx mori*, *Danaus plexippus plexippus*, and *H*. *armigera*’s protein tyrosine phosphatases non-receptor (PTPNs) protein sequences linked to gene accession numbers by BLAST search, and aligned those sequences, then estimated a tree from the aligned sequences and showed the tree in an image way to convey the relevant information to others.

### Statistical method

All the assays were repeated at least three times. Statistical analysis was conducted using Student’s *t*-test for pairwise comparison or analysis of variance (ANOVA) for multiple comparisons. In the figures, asterisks represent significant differences (*P < 0.05, and ** *P*< 0.01), and different lowercase letters show significant differences (*P* < 0.05). The values are the mean ± SD. Data were analyzed using Image-J software (NIH, Bethesda, MD, USA) for western blot and immunofluorescence, and generated using GraphPad 7.0 (GraphPad Inc., La Jolla, CA, USA). Average weight curves were analyzed using GraphPad 7.0 with thirty larvae each time and repeated three times.

## Supporting information

S1 Fig Western blotting to identify HaP60 and HaP110. **A.** The specificity of antibodies against HaP60, HaP110, and HaACTB using the fat body of 6th-48 h larvae. **B.** HaP60 and HaP110 detection from epidermis, midgut, and fat body of 6th-24 h larvae and P 6 d pupae. **C.** λPPase treatment to dephosphorylate HaP60 and HaP110 using 6th-48 h and 6th-96 h larval epidermis, midgut, and fat body. *P < 0.05 and **P < 0.01 using a two-tailed Student’s *t*-test. The bars indicate the mean ± SD.
(TIF)

S2 Fig Phylogenetic tree analysis of PTPNs from different species. *H*. *armigera* tyrosine phosphatase corkscrew-like isoform X1 (HaPTPN6), labeled with red, was adjacent with *D*. *plexippus* tyrosine-protein phosphatase non-receptor type 6 (PTPN6), and “100” in the branch presents the higher genetic relationship in evolution, therefore we named *H*. *armigera* tyrosine phosphatase corkscrew-like isoform X1 as *H*. *armigera* PTPN6 (HaPTPN6). *B*. *mori*, *Bombyx mori*; *D*. *plexippus*, *Danaus plexippus plexippus*; *D*. *melanogaster*, *Drosophila melanogaster*; *H*. *armigera*, *Helicoverpa armigera*; *H*. *sapiens*, *Homo sapiens*.
(TIF)

S3 Fig The efficiency of *HaPtpn6* knockdown was detected by qRT-PCR in HaEpi cells. *dsGFP* as control. *P < 0.05 using two-tailed Student’s *t*-test. The bars indicate mean ± SD.
(TIF)

S4 Fig Larvae fed with *E*. *coli* HT115 that expressed *dsHaPtpn6* and then injected *dsHaPtpn6* resulted in early pupation. **A.** Phenotype analysis after feeding *dsHaPtpn6* for 8 d and 10 d. **B.** Statistical analysis of early pupation in A by Student’s *t*-test based on three repeats. Thirty larvae for each repeat. **C.** The pupation time from 6th-0 h to pupa 0 d. **D.** The average pupal weight from the third instar feeding (3F) larvae to pupae 1 day (P-1 d) by Student’s *t*-test based on three repeats. Thirty larvae for each repeat. **E.** Efficiency analysis of *HaPtpn6* knockdown using qRT-PCR at the mRNA level. **F.** Morphology of midgut after feeding *dsRNA* for 8 d. MG: midgut. Bar, 0.5 cm. **G and H.** HE staining of midgut and fat body after feeding *dsRNA* for 8 d. LM: larval midgut; IM: imaginal midgut. Bars, 20 μm. *P < 0.05 and **P < 0.01 using two-tailed Student’s *t*-test. The bars indicate the mean ± SD.
(TIF)

S5 Fig 20E upregulated *HaPtpn6* expression through ErGPCRs- and EcR- signaling axis. **A.** *HaPtpn6* expression profiles in epidermis, midgut, and fat body at the mRNA level from 5F to A-0 day. **B and C.** 20E promoted *HaPtpn6* depended on dose and time manner in the fat body**. D and E.** Insulin did not affect *HaPtpn6* expression depended on time and dose in the fat body. **F-I.** *HaPtpn6* detection after *ErGpcr1*, *ErGpcr2*, *EcR* and *Usp1* knockdown in HaEpi cells. **J.** ChIP assay was conducted by transfecting EcR-RFP-His into HaEpi cells and treated with 20E induction. *P < 0.05 and **P < 0.01 using two-tailed Student’s *t*-test or different lowercase letters indicate significant differences (*P* < 0.05) using one-way ANOVA. The bars indicate mean ± SD.
(TIF)

S6 Fig *HaP60* and *HaP110* knockdown efficiency detection. **A.** The interference efficiency of *HaP60* and *HaP110* was detected by qRT-PCR at the mRNA level. **B and C.** The interference efficiency of *HaP60* and *HaP110* was detected by western blotting at the protein level. *P < 0.05 and **P < 0.01 using two-tailed Student’s *t*-test or different lowercase letters indicate significant differences (*P* < 0.05) using one-way ANOVA. The bars indicate mean ± SD.
(TIF)

S7 Fig RFP-His, HaP60-RFP-His, and HaP110-RFP-His detection by western blotting. (TIF)

S1 Table Primers used in the experiments. (DOCX)

S2 Table GenBank numbers of genes. (DOCX)
