# Supplementary figures and images for: Subunit P60 of phosphatidylinositol 3-kinase promotes cell proliferation or apoptosis depending on its phosphorylation status
Source: PLoS Genet. 2021 Apr 26;17(4):e1009514. doi: 10.1371/journal.pgen.1009514 (PMC8075199; doi:10.1371/journal.pgen.1009514)

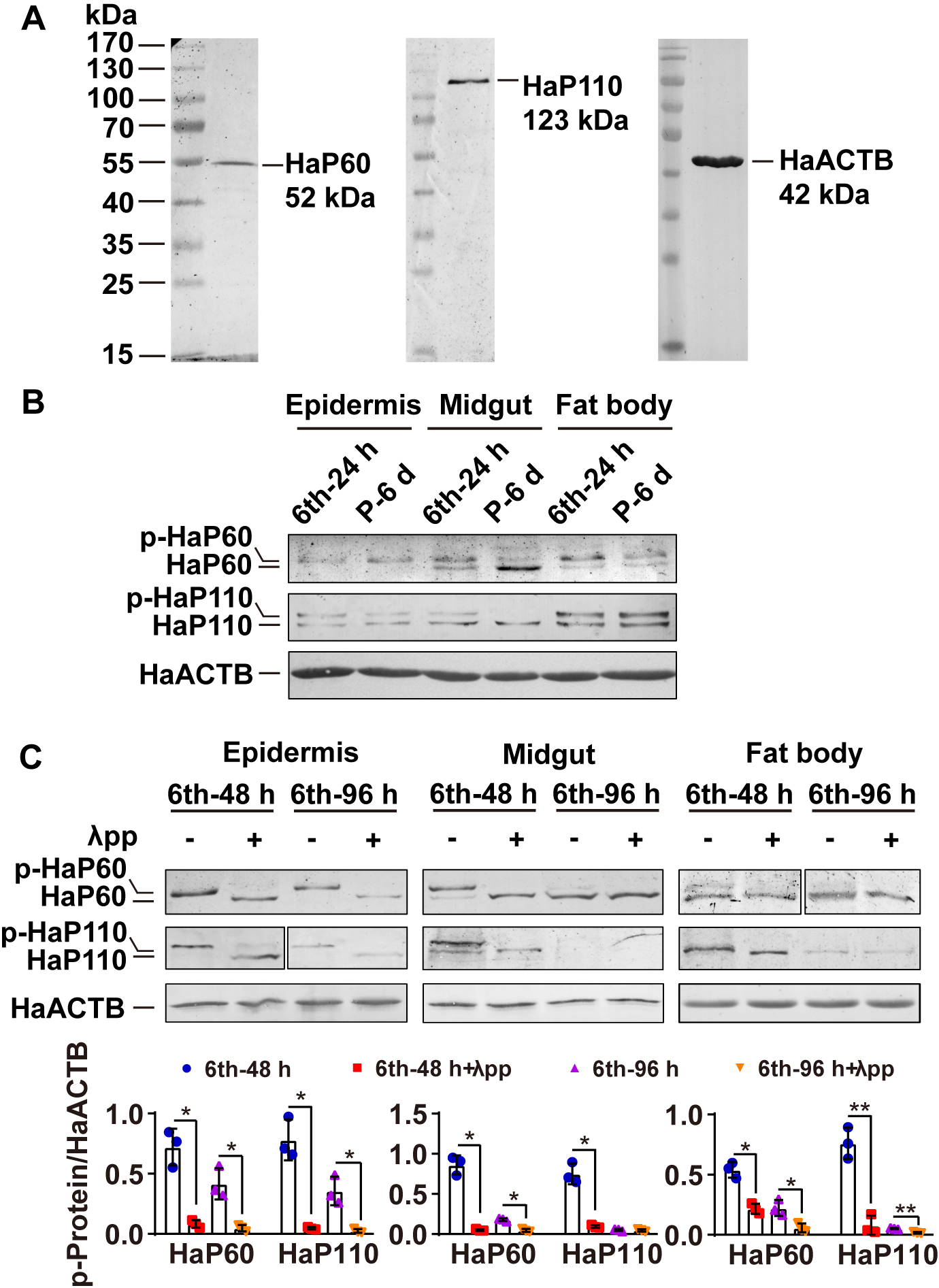

Supplement: S1 Fig — A. The specificity of antibodies against HaP60, HaP110, and HaACTB using the fat body of 6th-48 h larvae. B. HaP60 and HaP110 detection from epidermis, midgut, and fat body of 6th-24 h larvae and P 6 d pupae. C. λPPase treatment to dephosphorylate HaP60 and HaP110 using 6th-48 h and 6th-96 h larval epidermis, midgut, and fat body. *P < 0.05 and **P < 0.01 using a two-tailed Student’s t-test. The bars indicate the mean ± SD. (TIF) [file pgen.1009514.s001.tif]

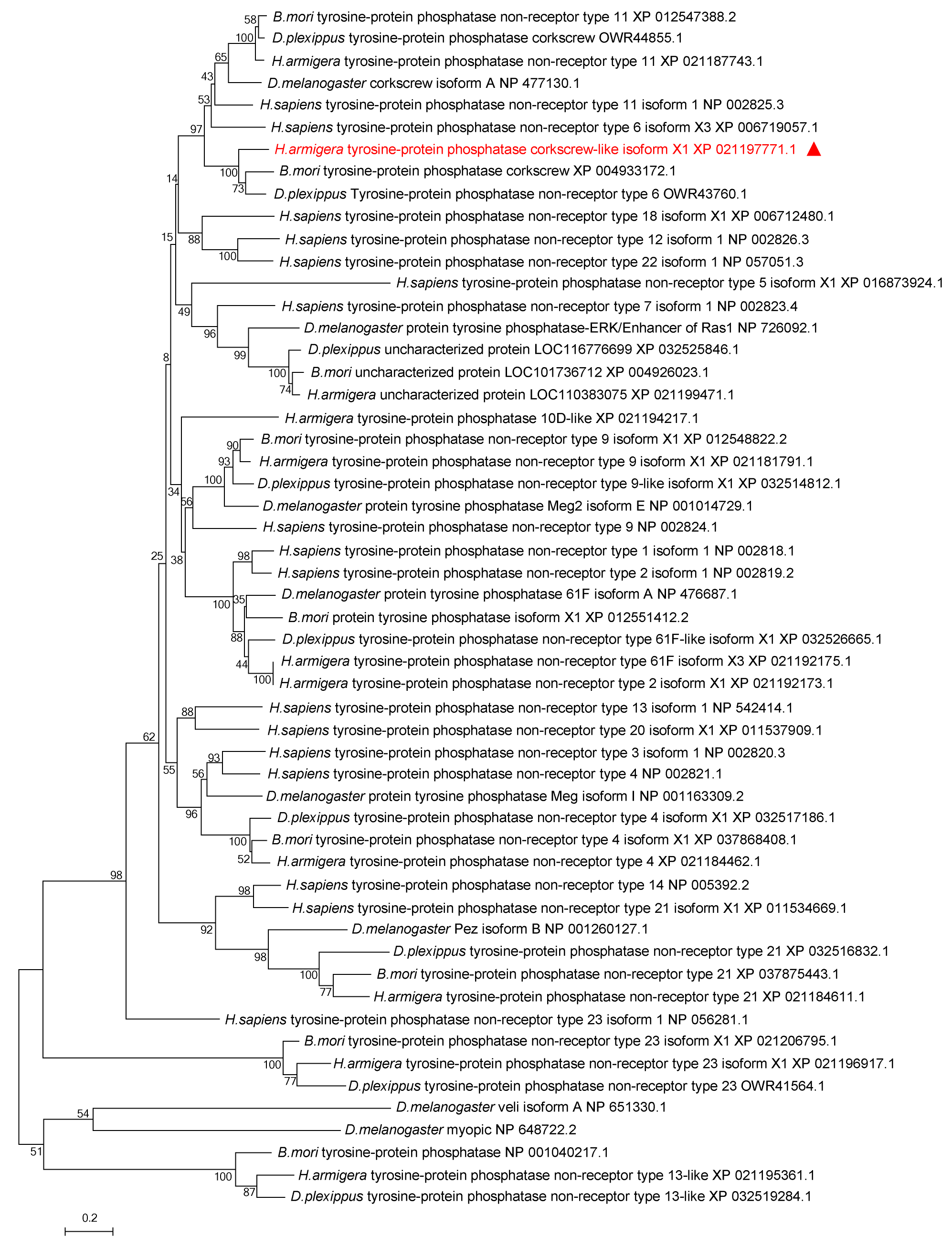

Supplement: S2 Fig — H. armigera tyrosine phosphatase corkscrew-like isoform X1 (HaPTPN6), labeled with red, was adjacent with D. plexippus tyrosine-protein phosphatase non-receptor type 6 (PTPN6), and “100” in the branch presents the higher genetic relationship in evolution, therefore we named H. armigera tyrosine phosphatase corkscrew-like isoform X1 as H. armigera PTPN6 (HaPTPN6). B. mori, Bombyx mori; D. plexippus, Danaus plexippus plexippus; D. melanogaster, Drosophila melanogaster; H. armigera, Helicoverpa armigera; H. sapiens, Homo sapiens. (TIF) [file pgen.1009514.s002.tif]

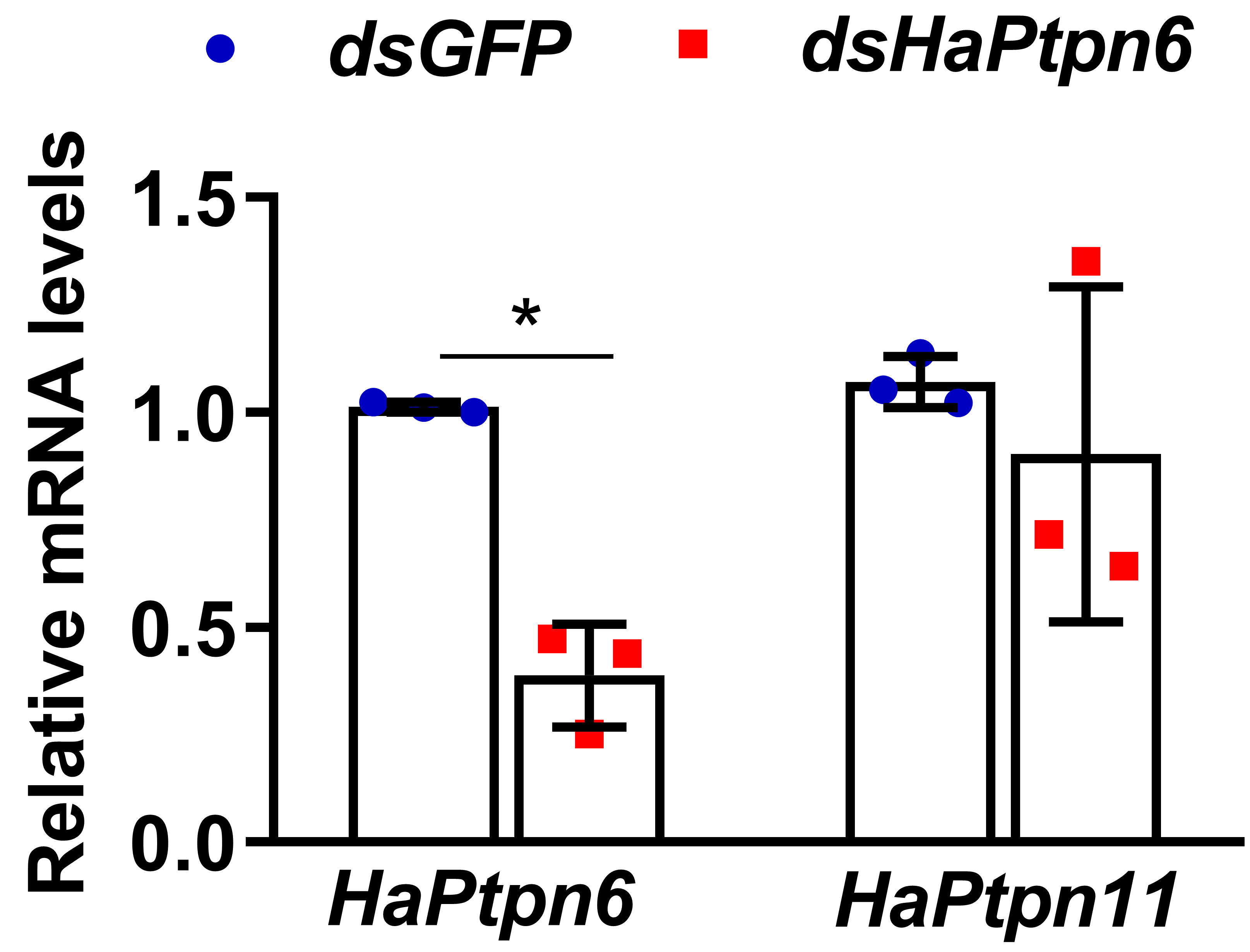

Supplement: S3 Fig — dsGFP as control. *P < 0.05 using two-tailed Student’s t-test. The bars indicate mean ± SD. (TIF) [file pgen.1009514.s003.tif]

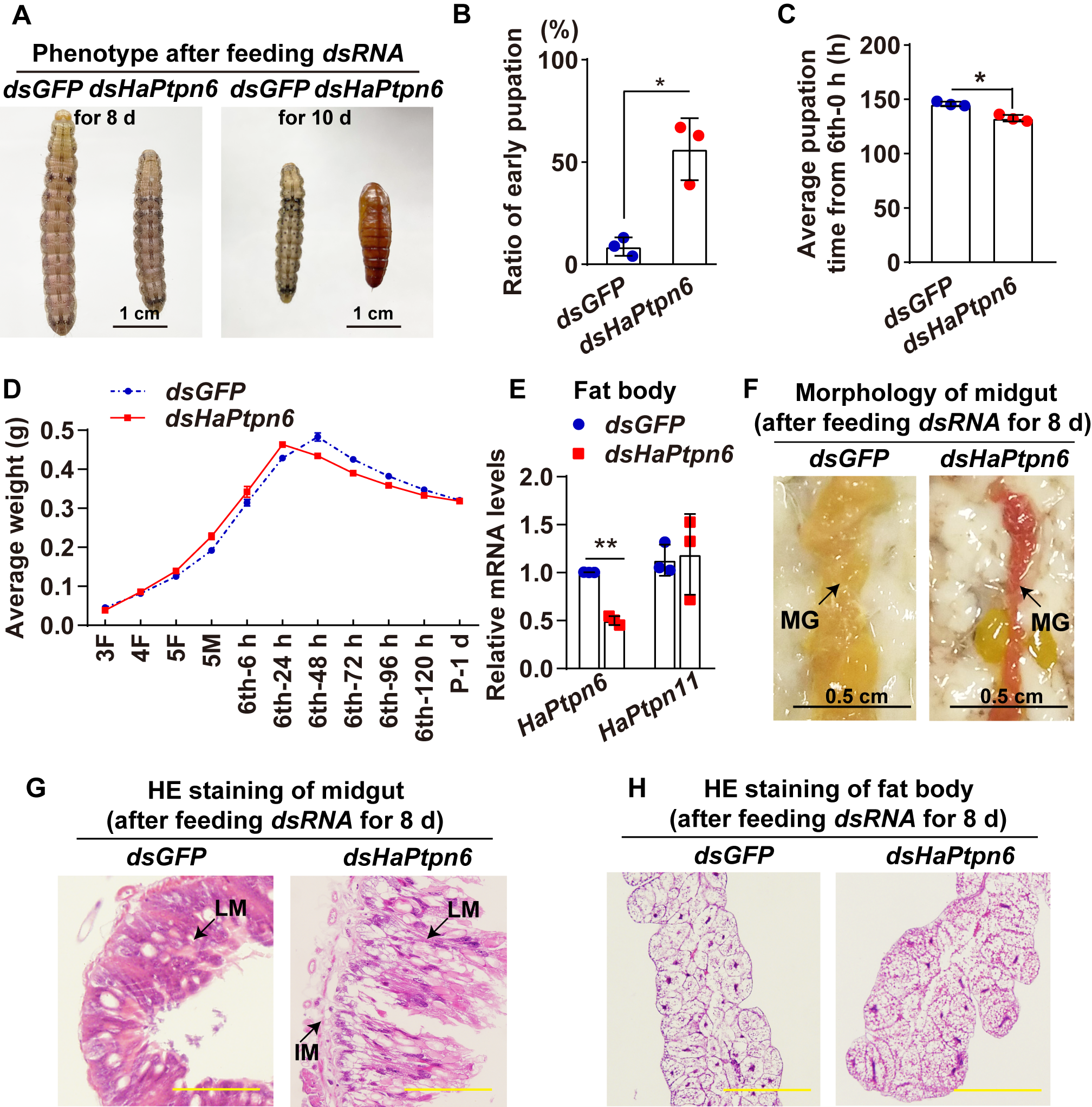

Supplement: S4 Fig — A. Phenotype analysis after feeding dsHaPtpn6 for 8 d and 10 d. B. Statistical analysis of early pupation in A by Student’s t-test based on three repeats. Thirty larvae for each repeat. C. The pupation time from 6th-0 h to pupa 0 d. D. The average pupal weight from the third instar feeding (3F) larvae to pupae 1 day (P-1 d) by Student’s t-test based on three repeats. Thirty larvae for each repeat. E. Efficiency analysis of HaPtpn6 knockdown using qRT-PCR at the mRNA level. F. Morphology of midgut after feeding dsRNA for 8 d. MG: midgut. Bar, 0.5 cm. G and H. HE staining of midgut and fat body after feeding dsRNA for 8 d. LM: larval midgut; IM: imaginal midgut. Bars, 20 μm. *P < 0.05 and **P < 0.01 using two-tailed Student’s t-test. The bars indicate the mean ± SD. (TIF) [file pgen.1009514.s004.tif]

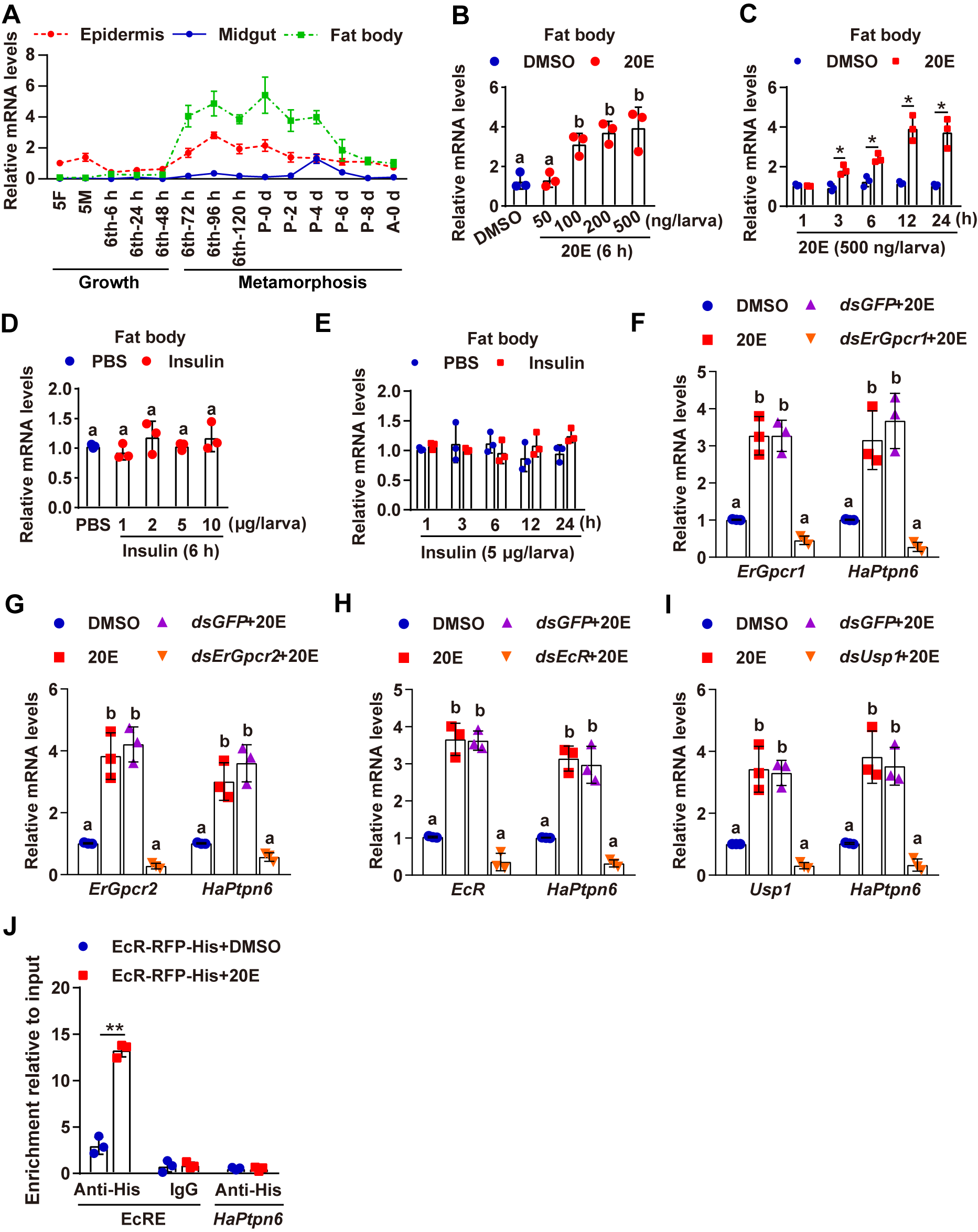

Supplement: S5 Fig — A. HaPtpn6 expression profiles in epidermis, midgut, and fat body at the mRNA level from 5F to A-0 day. B and C. 20E promoted HaPtpn6 depended on dose and time manner in the fat body. D and E. Insulin did not affect HaPtpn6 expression depended on time and dose in the fat body. F-I. HaPtpn6 detection after ErGpcr1, ErGpcr2, EcR and Usp1 knockdown in HaEpi cells. J. ChIP assay was conducted by transfecting EcR-RFP-His into HaEpi cells and treated with 20E induction. *P < 0.05 and **P < 0.01 using two-tailed Student’s t-test or different lowercase letters indicate significant differences (P < 0.05) using one-way ANOVA. The bars indicate mean ± SD. (TIF) [file pgen.1009514.s005.tif]

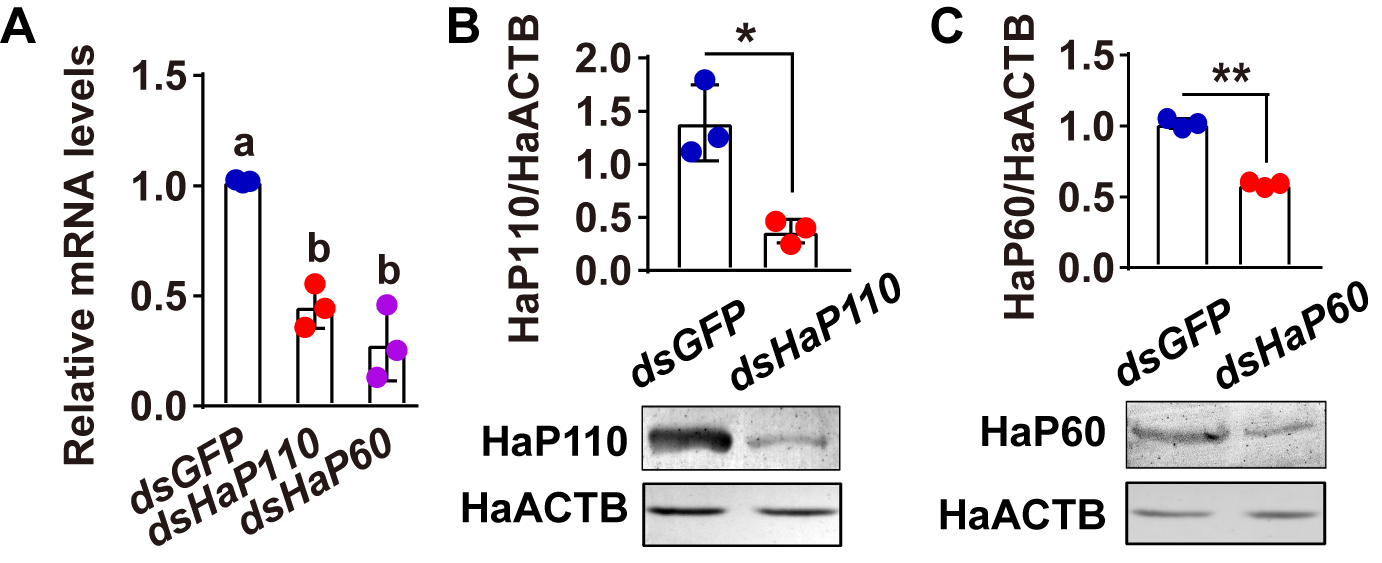

Supplement: S6 Fig — A. The interference efficiency of HaP60 and HaP110 was detected by qRT-PCR at the mRNA level. B and C. The interference efficiency of HaP60 and HaP110 was detected by western blotting at the protein level. *P < 0.05 and **P < 0.01 using two-tailed Student’s t-test or different lowercase letters indicate significant differences (P < 0.05) using one-way ANOVA. The bars indicate mean ± SD. (TIF) [file pgen.1009514.s006.tif]

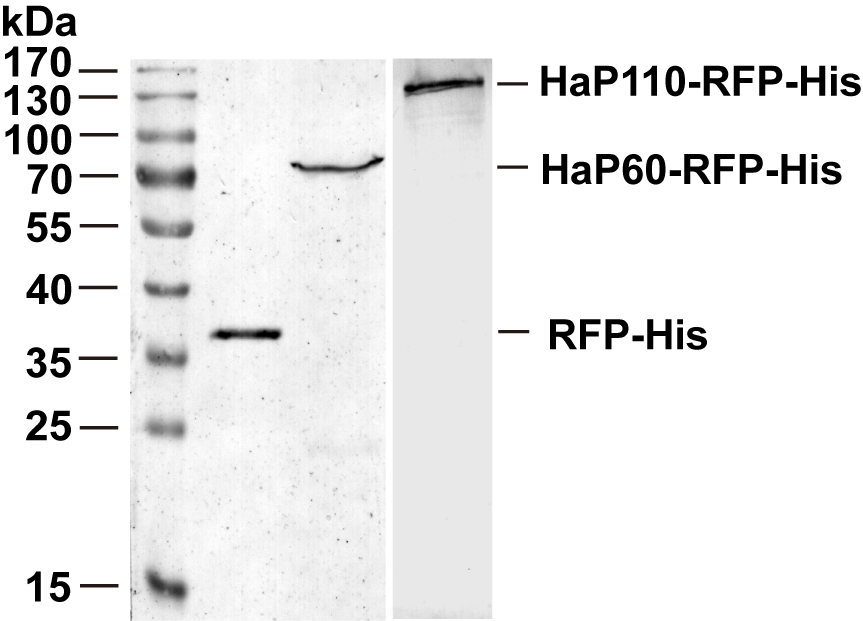

Supplement: S7 Fig — (TIF) [file pgen.1009514.s007.tif]
