# Supplementary material for: Subunit P60 of phosphatidylinositol 3-kinase promotes cell proliferation or apoptosis depending on its phosphorylation status
Source: PLoS Genet. 2021 Apr 26;17(4):e1009514. doi: 10.1371/journal.pgen.1009514 (PMC8075199; doi:10.1371/journal.pgen.1009514)
Supplement: S1 Table — (DOCX) [file pgen.1009514.s008.docx]

**S1 Table. Primers used in the experiments**

| Primer names | Primer sequence (5’-3’) |
| --- | --- |
| **Gene cloning** |  |
| *HaP60* F | TGTTAGCTCGTGTTGACTC |
| *HaP60* R | GTCGTCCTTTGACTTTGGA |
| *HaP110* F1 | ATGAAAAGTTGGGGACAGC |
| *HaP110* R1 | GTGTTACGGCTCACTTTCG |
| *HaP110* F2 | CGCAAGTTATCAGTGAAAGT |
| *HaP110* R2 | ACTGAAGCAAGTTTACCGT |
| *HaPtpn6* F | CGTAATAGTGCAAGCGCCAC |
| *HaPtpn6* R | GTCCAGTTATGTTTGCGATGAGT |
| **Expression** |  |
| *HaP60* exF | GAATCCAGGGTACCATGGTTGAAATAGGCGCCCCT |
| *HaP60* exR | GAATCCAGGGATCCTCATTGCTTTTTCTCAGGTTTC |
| *HaP110* exF | TACTCAACGGTACCACAGTGAAAGATTACTCGG |
| *HaP110* exR | TACTCAACCTCGAGTCCACTTAAGCTTATCCAGG |
| **QRT-PCR** |  |
| *HaActb* QRTF | CCTGGTATTGCTGACCGTATGC |
| *HaActb* QRTR | CTGTTGGAAGGTGGAGAGGGAA |
| *EcR* QRTF | AATTGCCCGTCAGTACGA |
| *EcR* QRTR | TGAGCTTCTCATTGAGGA |
| *ErGpcr1* QRTF | AAACGGTTCACCTACTACGC |
| *ErGpcr1* QRTR | CGCTTCATCTTCGCTATCT |
| *ErGpcr2* QRTF | CGAGGGTCAAGTCTGAGGTT |
| *ErGpcr2* QRTR | TATTATTAGTCGTGGTGGTA |
| *HaFoxo* QRTF | TCATTACCCAAGCCAGCAC |
| *HaFoxo* QRTR | TCCATCCAGCCGAAGAGT |
| *HaP60* QRTF | GGATTTGCCGAACCCTATA |
| *HaP60* QRTR | ACAAGATGTGCGTTTACTG |
| *HaP110* QRTF | TGGAGGAGTTCACGATGA |
| *HaP110* QRTR | CCCTTCTGTCCCTTATTG |
| *HaPtpn6* QRTF | TGTCACCAAGTCGCTGTC |
| *HaPtpn6* QRTR | ATCCTCCTGCCATAACATCC |
| *HaPtpn11* QRTF | CTTCTTAGCAAGACCCAGCAT |
| *HaPtpn11* QRTR | AGGTCAAGGAACTCGCCATT |
| *Usp1* QRTF | GGTCCTGACAGCAATGTT |
| *Usp1* QRTR | TTCCAGCTCCAGCTGACTGAAG |
| **RNAi** |  |
| *EcR* RNAiF | GAGTAATACGACTCACTATAGGGACGCTGGTATAACAACGGAGGA |
| *EcR* RNAiR | GAGTAATACGACTCACTATAGGGAAGCTGGAGCAACTCCTCACG |
| *ErGpcr1* RNAiF | GCGTAATACGACTCACTATAGGGTTCATCCTTCTAACGGTGGC |
| *ErGpcr1* RNAiR | GCGTAATACGACTCACTATAGGGTCGCTTCATCTTCGCTATCT |
| *ErGpcr2* RNAiF | GCGTAATACGACTCACTATAGGCGAGGGTCAAGTCTGAGGTT |
| *ErGpcr2* RNAiR | GCGTAATACGACTCACTATAGGTTAAGGCTGTTTGATGTTGA |
| *HaFoxo* RNAiF | GCGTAATACGACTCACTATAGAACTCTTCGGCTGGATGGA |
| *HaFoxo* RNAiR | GCGTAATACGACTCACTATAGGTGCGTGCTGATGTGGGT |
| *HaP60* RNAiF | GCGTAATACGACTCACTATAGATGGCACCTTCTTGG |
| *HaP60* RNAiR | GCGTAATACGACTCACTATAGACCTCGCCCTTGTAG |
| *HaP110* RNAiF | GCGTAATACGACTCACTATAGTCAGCGACAACGACCTACT |
| *HaP110* RNAiR | GCGTAATACGACTCACTATAGCACCTCGATCATACCCACC |
| *HaPtpn6* RNAiF | GCGTAATACGACTCACTATAGCATGGTGTAATGTCAGCTA |
| *HaPtpn6* RNAiR | GCGTAATACGACTCACTATAGTGTTCAAGATCAGACAGT |
| *Usp1* RNAiF | GAGTAATACGACTCACTATAGGGACGAACCATCCCCTAAGTGGTTC |
| *Usp1* RNAiR | GAGTAATACGACTCACTATAGGGACCTTGATGAGCAGGATCTGGTC |
| **Overexpression** |  |
| *HaP60* oexF | GAAGATCTCGATGGTTGAAATAGGCGCCC |
| *HaP60* oexR | GGGGTACC TTGCTTTTTCTCAGGTTTCTTCAC |
| *HaP110* oexF | GAAGATCTCGATGGTTCCTGCGCCGA |
| *HaP110* oexR | TTGGCGCGCCGAGTTGTTCTTGTCGATGTTGTGAAT |
| *HaPten* oexF | GAAGATCTCGATGGGTATTTGCGTGAGC |
| *HaPten* oexR | TTGGCGCGCCGACAAGTATGTAGATTCACC |
| *HaPtpn6* oexF | GAAGATCTCG ATGTCCCGCGAGAGCCG |
| *HaPtpn6* oexR | TTGGCGCGCCGATGTTTGCGATGAGTTTACC |
| **Feeding exp** |  |
| *HaP60* F | TACTCAGAGCTCCTGAAGCACAGCCACAT |
| *HaP60* R | TACTCACTCGAGGTTCGGCAAATCCATAA |
| *HaP110* F | TACTCAAGATCTGGAGGAAGTGGACGACG |
| *HaP110* R | TACTCACCCGGGGGGACGCTTGATGAGAA |
| *HaPtpn6* F | TACTCAGAGCTCCATGGTGTAATGTCAGCTA |
| *HaPtpn6* R | TACTCACTCGAGGTTCAAGATCAGACAGT |
| **ChIP** |  |
| *HaP60* PF | CCTGAATTTGATGGTGGAAA |
| *HaP60* PR | AAACTTGCATCTCGTTTGCG |
| *HaPtpn6* PF | TCAATGACTCTCAACGCA |
| *HaPtpn6* PR | ACTTTGCACTCTTGTATAAC |
|  | |
