# Supplementary material for: Subunit P60 of phosphatidylinositol 3-kinase promotes cell proliferation or apoptosis depending on its phosphorylation status
Source: PLoS Genet. 2021 Apr 26;17(4):e1009514. doi: 10.1371/journal.pgen.1009514 (PMC8075199; doi:10.1371/journal.pgen.1009514)
Supplement: S2 Table — (DOCX) [file pgen.1009514.s009.docx]

| Genes Name | GenBank numbers of genes | Name | GenBank numbers of genes |
| --- | --- | --- | --- |
| *HaP60* | XM_021326574.1 | *HaFoxo* | XM_021330987.1 |
| *HaP110* | XM_021329589.1 | *ErGpcr1* | JQ809653.1 |
| *HaPtpn6* | XM_021342096.1 | *ErGpcr2* | AKA95280.1 |
| *HaPtpn11* | XM_021332068.1 | *EcR* | ACD74807.1 |
| *HaPten* | XM_021330464.1 | *Usp1* | ACD74808.1 |

**S2 Table. GenBank numbers of genes**
